# Deep mitigation of CO_2_ and non-CO_2_ greenhouse gases toward 1.5 °C and 2 °C futures

**DOI:** 10.1038/s41467-021-26509-z

**Published:** 2021-10-29

**Authors:** Yang Ou, Christopher Roney, Jameel Alsalam, Katherine Calvin, Jared Creason, Jae Edmonds, Allen A. Fawcett, Page Kyle, Kanishka Narayan, Patrick O’Rourke, Pralit Patel, Shaun Ragnauth, Steven J. Smith, Haewon McJeon

**Affiliations:** 1 Joint Global Change Research Institute, Pacific Northwest National Laboratory, College Park, MD USA; 2 U.S. Environmental Protection Agency, Washington, DC USA; 3 Present Address: Electric Power Research Institute, Washington, DC USA

**Keywords:** Climate-change mitigation, Climate-change mitigation

## Abstract

Stabilizing climate change well below 2 °C and towards 1.5 °C requires comprehensive mitigation of all greenhouse gases (GHG), including both CO_2_ and non-CO_2_ GHG emissions. Here we incorporate the latest global non-CO_2_ emissions and mitigation data into a state-of-the-art integrated assessment model GCAM and examine 90 mitigation scenarios pairing different levels of CO_2_ and non-CO_2_ GHG abatement pathways. We estimate that when non-CO_2_ mitigation contributions are not fully implemented, the timing of net-zero CO_2_ must occur about two decades earlier. Conversely, comprehensive GHG abatement that fully integrates non-CO_2_ mitigation measures in addition to a net-zero CO_2_ commitment can help achieve 1.5 °C stabilization. While decarbonization-driven fuel switching mainly reduces non-CO_2_ emissions from fuel extraction and end use, targeted non-CO_2_ mitigation measures can significantly reduce fluorinated gas emissions from industrial processes and cooling sectors. Our integrated modeling provides direct insights in how system-wide all GHG mitigation can affect the timing of net-zero CO_2_ for 1.5 °C and 2 °C climate change scenarios.

## Abstract (web summary)

Stabilizing climate change requires simultaneous mitigation of all greenhouse gas emissions (GHGs). Here the authors examine 90 mitigation scenarios pairing different levels of CO_2_ and non-CO_2_ GHG abatement pathways to demonstrate the contributions of different GHGs towards 1.5 °C and 2 °C goals.

## Introduction

The Paris Agreement aims to strengthen the global response to climate change mitigation by keeping a global temperature rise this century well below 2 °C above pre-industrial levels and “pursue efforts to limit the temperature increase to 1.5 °C” (Article 2). Achieving these goals implies a tight limit on cumulative greenhouse gas (GHGs) emissions, which should “reach global peaking of GHG emissions as soon as possible”, followed by rapid reductions “to achieve a balance between anthropogenic emissions by sources and removals by sinks of greenhouse gases in the second half of this century” (Article 4). The emission trajectories are consistent with the recent net-zero emissions pledges, including China’s 2060 net-zero pledge^1^, EU’s 2050 climate neutrality pledge^2^, and California’s 2045 carbon neutrality pledge^3^. Recent studies have emphasized the importance and urgency of reaching net-zero CO_2_ emissions to achieve stringent climate targets^4–7^.

Modeling scenarios avoiding global warming >1.5 °C, as stipulated in the Paris Agreement, requires combined mitigation of CO_2_ and non-CO_2_ GHG emissions^8,9^. Non-CO_2_ GHGs accounted for about one quarter of total CO_2_-eq emissions in 2015^10^. Thus, cuts in their emissions could potentially lessen future climate forcing^11^. The present literature indicates that the ultimate level of surface temperature warming depends both on driving CO_2_ emissions to zero but also on the residual level of non-CO_2_ emissions^5,9,12–20^. Although these studies have in some way accounted for the climate benefits for non-CO_2_ mitigation, different representations and non-CO_2_ mitigation options, as well as the economic structure of the models, can lead to a fairly large variation in the reported remaining carbon budget or net-zero commitment years that aim to achieve the same 1.5 °C or 2 °C goals^21–23^.

A comprehensive evaluation of the climate benefits for system-wide non-CO_2_ GHG mitigation is challenging due to the difficulties of identifying and parameterizing the numerous possible mitigation options for different gases emitted from various sectors. In addition, some CO_2_ mitigation and non-CO_2_ mitigation actions are intertwined across sectors^24^. For example, phasing out fossil fuels can both reduce direct CO_2_ emissions from fuel combustion and upstream methane emissions from fossil fuel extraction. Furthermore, the techno-economic mitigation potential for each source and mitigation technology is also evolving, affected by technology innovation. These dynamics can further complicate the comprehensive non-CO_2_ representation in long-term mitigation analysis. Therefore, a robust analysis of all GHG mitigation must combine sectoral and regional detail of mitigation data with an integrated representation of the entire economic system, including energy, industrial processes, buildings, transport, urban process, and agriculture sectors.

Here we combine the latest region-, sector-, and year-specific EPA non-CO_2_ abatement datasets with the Global Change Analysis Model (GCAM)^25^ to explore how CO_2_ and non-CO_2_ GHG mitigation pathways jointly affect the ultimate level of temperature change. Specifically, we investigate to which degree a comprehensive non-CO_2_ mitigation scheme covering all GHGs (CH_4_, N_2_O, HFCs, PFCs, and SF_6_) (Supplementary Table 1) and all sectors (energy, industrial processes, buildings, transport, urban process, and agriculture) (Supplementary Table 2) can affect the timing of net-zero CO_2_ required to achieve 1.5 °C and 2 °C targets. We construct 90 mitigation scenarios pairing 30 CO_2_ abatement pathways with three non-CO_2_ abatement levels to evaluate the mitigation potential of each non-CO_2_ GHG to the overall stabilization efforts (Supplementary Fig. 1) through both reduced fossil fuel use and adoption of specific non-CO_2_ abatement measures endogenously driven by societal carbon prices. Our research aims at better disentangling and assessing the interactions between non-CO_2_ GHG mitigation and net-zero CO_2_ commitments toward 1.5 °C and 2 °C goals and is an extension of the conceptual framework in ref. ^22^.

## Results

### Effects of non-CO_2_ abatement on climate response

**Fig. 1:**
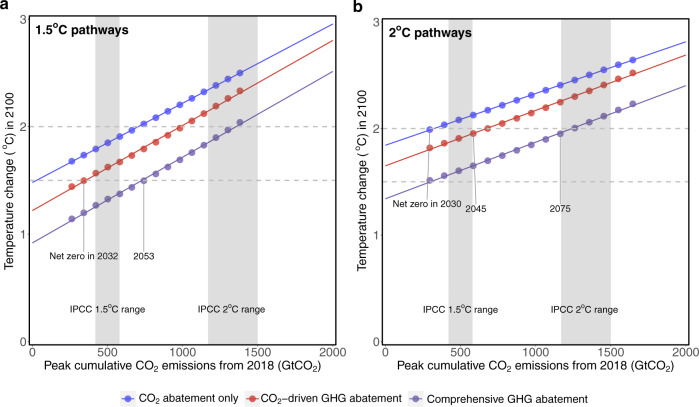
Transient climate response to cumulative emissions of CO_2_ for different non-CO_2_ abatement levels. **a** 1.5 °C pathways and (**b**) 2 °C pathways, assume CO_2_ emissions linearly reduce to −8 and 0 GtCO_2_ yr^−1^ and stay constant, correspondingly. CO_2_ abatement only assumes the same non-CO_2_ emissions as the Reference scenario while reducing CO_2_ emissions alone; CO_2_-driven GHG abatement includes CO_2_ abatement and the non-CO_2_ abatement associated with fuel switching and demand reduction driven by CO_2_ abatement; Comprehensive GHG abatement additionally considers various non-CO_2_ abatement options based on their technical feasibility and cost-effectiveness. For (**a**) net-zero years are interpolated based on the linear CO_2_ emission trajectories.

We developed 90 mitigation scenarios by combining 30 alternative years in which global net-zero CO_2_ emission levels are achieved with three levels of non-CO_2_ abatement. The CO_2_ abatement pathways are split into two levels of negative CO_2_ assumptions. The 2 °C pathways linearly reach net-zero CO_2_ emissions between 2030 and 2100 in 5-year increments, followed by zero CO_2_ emissions afterwards, and the 1.5 °C pathways linearly reach −8 GtCO_2_ yr^−1^ CO_2_ emissions between 2030 and 2100 in 5-year increments (so their net-zero CO_2_ years are slightly earlier), followed by −8 GtCO_2_ yr^−1^ CO_2_ emissions afterwards. The endpoints of 0 and −8 GtCO_2_ yr^−1^ CO_2_ are generally on the conservative side of negative CO_2_ emissions required for 2 °C and 1.5 °C pathways^26^, respectively. For non-CO_2_ GHG mitigation, CO_2_ abatement only assumes the same non-CO_2_ emissions as the Reference scenario while reducing CO_2_ emissions alone, which is a counterfactual scenario aiming at isolating the climate impact of CO_2_ mitigation alone. CO_2_-driven GHG abatement includes CO_2_ abatement and the non-CO_2_ emission reductions associated with fuel switching and demand reduction driven by CO_2_ abatement. Comprehensive GHG abatement additionally includes specific non-CO_2_ abatement measures driven by increased societal carbon prices. Figure 1 illustrates how non-CO_2_ abatement levels are combined with carbon budgets to jointly determine the end-of-century temperature changes.

The temperature increase (°C) since pre-industrial times is linearly associated with the remaining carbon budget^27^, consistent with literature^22^, which is determined by peak cumulative CO_2_ emissions and in this analysis, the timing of the year in which net-zero CO_2_ emissions are achieved. The transient climate response to cumulative emissions of CO_2_ are similar across different non-CO_2_ abatement levels, falling within the IPCC reported range of 0.2–0.7 °C per 1000 Gt CO_2_ (66% probability)^27^. However, the ultimate level of temperature change is jointly affected by both the timing of net-zero CO_2_ and the stringency of non-CO_2_ GHG abatement. In Comprehensive GHG abatement scenarios, the timing of net-zero CO_2_ could be two decades later to achieve the same temperature change levels, compared with CO_2_-driven GHG abatement that only considers emission reductions from fuel switching and service demand reduction (Table 1).

**Table 1:** Net-zero CO_2_ emission years necessary to achieve 1.5 °C and 2 °C future.

Mitigation pathway	1.5 °C	2 °C
CO_2_ abatement only	Unsolvable	2030
CO_2_-driven GHG abatement	2032	2045
Comprehensive GHG abatement	2053	2075

In 1.5 °C pathways, CO_2_ emissions linearly reduce to −8 GtCO_2_ yr^−1^ and stay constant. In 2 °C pathways, CO_2_ emissions linearly reduce to 0 GtCO_2_ yr^−1^ and stay constant.

**Fig. 2:**
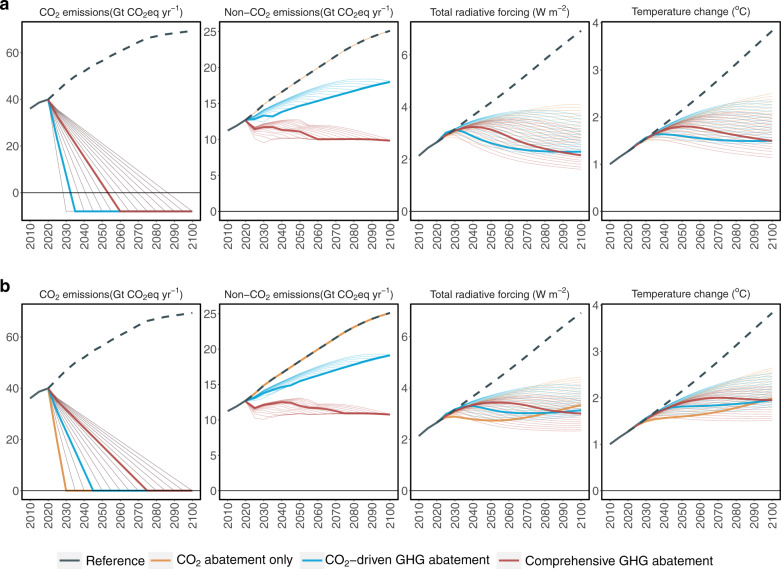
Global emission and climate results. **a** 1.5 °C and (**b**) 2 °C scenarios, including total CO_2_ emissions from fossil fuels and land-use changes, total non-CO_2_ GHG (CH_4_, N_2_O, HFCs, P.F.C.s, and SF_6_) emissions, total radiative forcing, and global mean surface temperature change. Reference assumes no GHG mitigation. CO_2_ abatement only cannot achieve the 1.5 °C target under all modeled 1.5 °C pathways but achieves the 2 °C target if reaching net-zero CO_2_ by 2030 under 2 °C pathways; CO_2_-driven GHG abatement achieves the 1.5 °C target if reaching net-zero CO_2_ by 2032 under 1.5 °C pathways or achieves the 2 °C target if reaching net-zero CO_2_ by 2045 under 2 °C pathways; Comprehensive GHG abatement achieves the 1.5 °C target if reaching net-zero CO_2_ by 2053 under 1.5 °C pathways or achieves the 2 °C target if reaching net-zero CO_2_ by 2075 under 2 °C pathways. The faint lines are colored based on non-CO_2_ mitigation pathways, representing scenarios with CO_2_ emission constraints reaching net-zero in alternative years. Non-CO_2_ GHG emissions were aggregated with GWP-100 from ref. ^28^.

Among all 90 mitigation scenarios explored, the same 1.5 °C and 2 °C targets can be achieved with different combinations of net-zero CO_2_ commitment and non-CO_2_ abatement levels (thick lines in Fig. 2). Per 2007 IPCC Fourth Assessment Report (AR4)^28^, Global Warming Potentials (GWPs) based on a timeframe of 100 years (GWP-100) are used to estimate CO_2_-eq emissions for non-CO_2_ GHGs. In Reference, global CO_2_ and non-CO_2_ GHG emissions continue to grow throughout the century, reaching 69 Gt CO_2_-eq yr^−1^ and 25 Gt CO_2_-eq yr^−1^, respectively, driven by increasing population and GDP. The mean temperature increases by 3.8 °C by 2100. In 1.5 °C scenarios (Fig. 2a), the CO_2_ abatement only is infeasible, and CO_2_-driven GHG abatement requires net-zero CO_2_ emissions to be reached by ~2032 (and −8 GtCO_2_ yr^−1^ in 2035). The timing of reaching net-zero CO_2_ here emphasizes the urgency of transitioning to net-zero emissions for the 1.5 °C target^29^. In the CO_2_-driven GHG abatement scenario, non-CO_2_ emissions decrease by 28% relative to Reference, reaching 18 Gt CO_2_-eq yr^−1^ in 2100. However, non-CO_2_ emissions still show a growing trend in the CO_2_-driven GHG abatement scenario, suggesting a significant amount of residual non-CO_2_ emissions when reductions are driven solely by CO_2_ mitigation actions. In the comprehensive GHG mitigation scenario that further accounts for non-CO_2_ abatement measures, non-CO_2_ emissions show a decreasing trend, reducing to 9.8 Gt CO_2_-eq yr^−1^ in 2100. As a result, the 1.5 °C target is viable by reaching net-zero CO_2_ in ~2053 (and −8 GtCO_2_ yr^−1^ in 2060). Figure 2b shows the same results for the 2 °C scenarios, in which CO_2_ abatement only and CO_2_-driven abatement scenarios would need to reach net-zero CO_2_ by 2030 and 2045, respectively. In contrast, Comprehensive GHG abatement allows net-zero CO_2_ to be achieved by 2070 without jeopardizing the 2 °C goal. Notably, net negative CO_2_ emissions are not required in 2 °C scenarios.

We further conducted three sets of sensitivity analysis for the 1.5 °C scenario with comprehensive GHG mitigation (red line in Fig. 2a), additionally exploring three alternative technology change assumptions (Supplementary Fig. 2), five shared socioeconomic pathways (SSPs) (Supplementary Fig. 3), and four alternative GWP assumptions (Supplementary Figs. 4–6). While the 2019 EPA mitigation report^10^ estimates used here represent non-CO_2_ mitigation measures and technology innovation to 2050, assumptions of technology change after 2050 have limited impact on the end-of-century forcing and temperature changes (Supplementary Fig. 2). Even without technology changes after 2050, our comprehensive GHG mitigation scenario can still achieve the 1.5 °C target. However, the underlying socioeconomic pathways have a fairly significant impact (Supplementary Fig. 3), and some SSP scenarios would fail to achieve the 1.5 °C target, while all SSP scenarios can still achieve the well-below 2 °C target.

### Non-CO_2_ emission abatement actions

**Fig. 3:**
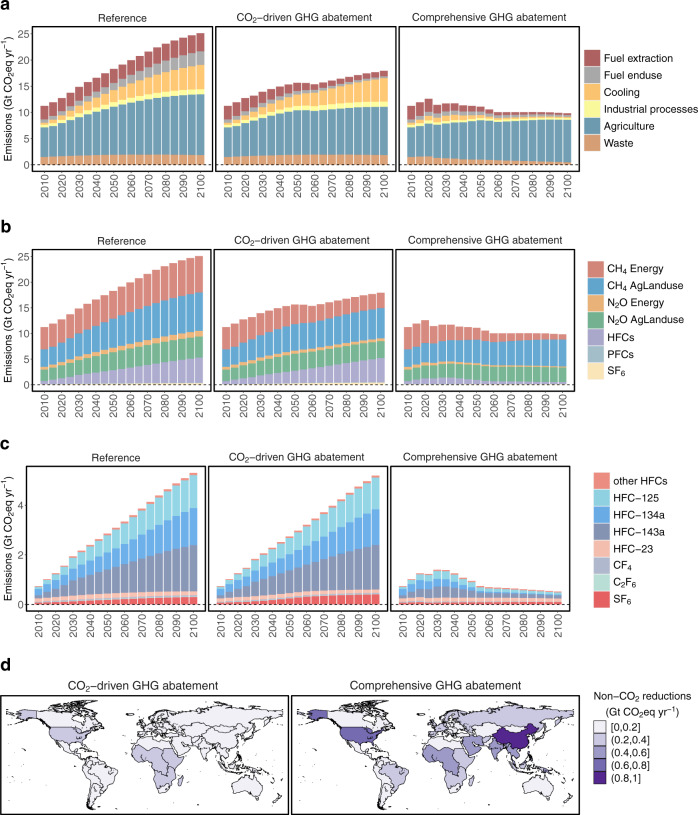
Global non-CO_2_ GHG emissions. Emissions by (**a**) sector and (**b**) species, (**c**) F-gas emissions by species, and (**d**) reductions in 2050, when reaching net-zero CO_2_ emissions by 2053 (and −8 GtCO_2_ in 2060) for a 1.5 °C scenario. Other HFCs include HFC-32, HFC-43, HFC-152a, HFC-227ea, HFC-236fa, HFC-245fa, and HFC-365mfc. Non-CO_2_ GHG emissions were aggregated with GWP-100, from ref. ^28^. Global maps in this figure are created using an open-source R package. ^55^ and documented in ref. ^56^.

Future non-CO_2_ reductions are determined by the level of CO_2_ mitigation as well as non-CO_2_ abatement measures. Here we focus on the CO_2_ mitigation pathways reaching net-zero by 2053, which achieves the 1.5 °C target in Comprehensive GHG abatement (Fig. 3). Without additional non-CO_2_ abatement measures, reductions in non-CO_2_ GHG emissions are achieved by cuts in fuel extraction and other energy sector activity due to the lower reliance on fossil fuels driven by CO_2_ mitigation (Supplementary Fig. 7). However, cooling-related HFC emissions and industrial process emissions (PFCs and SF_6_) are barely affected by fuel switching. Agriculture CH_4_ and N_2_O emissions from livestock population, rice cultivation, and fertilizer application, are also slightly reduced from the Reference (Supplementary Fig. 8) due to the overall demand reduction from decarbonization actions, which increases the overall prices of services and goods.

When additionally including system-wide non-CO_2_ mitigation measures in Comprehensive GHG abatement, industrial and cooling-related HFCs emissions are heavily reduced (Fig. 3a–b). In 2050, 68% of HFCs are reduced from the Reference, whereas only 6% are reduced in the CO_2_-driven GHG abatement scenario (Table 2). In 2100, 92% of HFCs are reduced from the Reference, leading to an 85% reduction in HFC forcings. HFCs are mainly used as alternatives to several classes of ozone-depleting substances required to be phased out under the Montreal Protocol. Among different HFC species, HFC134a, HFC143a, and HFC125 accounts for over 90% of the total F-gas emissions in both Reference and CO_2_-driven GHG abatement scenarios, while they are all effectively reduced in Comprehensive GHG abatement (Fig. 3c), accounting for a large share of total forcing reductions in Comprehensive GHG abatement (Supplementary Fig. 9). These HFCs are used in various industrial and building applications, including refrigeration and air-conditioning equipment, aerosols, solvent cleaning, fire extinguishing, foam production, and sterilization. These sources have considerable mitigation potential, such as leak repair for existing requirements, refrigerant recovery at disposal for existing refrigeration with AC equipment, and the adoption of other non-GHG cooling agents to replace current HFCs^10^. The Kigali Amendment to the Montreal Protocol requires global HFC emissions to be heavily reduced by 2050 relative to the present levels^30^. In Comprehensive GHG abatement, most regions continue to reduce their HFCs after 2050, and the average HFC reductions across all GCAM regions achieve 90% by 2065 relative to their present levels.

**Table 2:** Non-CO_2_ emission and climate forcing changes in mitigation scenarios relative to Reference in 2050 and 2100 when CO_2_ emissions reach net-zero by 2053 under 1.5 °C pathway (percentage reductions are shown in parentheses).

Scenario	CH_4_ Energy	CH_4_ AgLanduse	N_2_O Energy	N_2_O AgLanduse	HFCs	PFCs	SF_6_
2050 Emission (Gt CO_2_-eq yr^−1^)							
CO_2_-driven	−1.58 (−29.3%)	−0.51 (−8.9%)	−0.26 (−31.2%)	−0.19 (−5.3%)	−0.16 (−6.2%)	−0.01 (−15.1%)	0.06 (31.8%)
Comprehensive	−3.09 (−57.2%)	−1.10 (−19.2%)	−0.52 (−61.9%)	−0.59 (−16.8%)	−1.77 (−68.2%)	−0.04 (−53.4%)	−0.10 (−52.6%)
2100 Emission (Gt CO_2_-eq yr^-1^)							
CO_2_-driven	−4.11 (−57.8%)	−1.58 (−21.1%)	−0.57 (−52.1%)	−0.80 (−19.4%)	−0.20 (−4.1%)	−0.01 (−16.0%)	0.11 (36.0%)
Comprehensive	−6.10 (−85.8%)	−2.22 (−29.6%)	−0.91 (−83.4%)	−1.26 (−30.7%)	−4.52 (−91.7%)	−0.06 (−79.8%)	−0.20 (−67.9%)
2050 Forcing (W m^−2^)							
CO_2_-driven	−0.046 (−11.1%)	−0.001 (−0.3%)	−0.007 (−5.3%)	0.000 (0.0%)	−0.007 (−5.3%)	0.000 (−1.7%)	0.000 (6.4%)
Comprehensive	−0.128 (−31.0%)	−0.022 (−10.6%)	−0.020 (−14.3%)	−0.005 (−4.2%)	−0.062 (−46.3%)	−0.001 (−6.4%)	−0.002 (−23.6%)
2100 Forcing (W m^−2^)							
CO_2_-driven	−0.170 (−32.3%)	−0.034 (−12.5%)	−0.041 (−18.1%)	−0.019 (−10.5%)	−0.018 (−5.4%)	−0.001 (−5.1%)	0.005 (27.0%)
Comprehensive	−0.285 (−54.1%)	−0.067 (−24.9%)	−0.075 (−33.3%)	−0.037 (−20.7%)	−0.287 (−85.0%)	−0.003 (−21.1%)	−0.008 (−47.7%)

Non-CO_2_ GHG emissions were aggregated with GWP-100 from ref. ^28^.

SF_6_ emissions are even higher in the CO_2_-driven GHG abatement scenario than in Reference due to greater electrification. SF_6_ is mainly used in electrical transmission and distribution systems as an insulating medium. In Comprehensive GHG abatement, SF_6_ emission decreases by 68% in 2100 and its climate forcing decreases 48%. Note that SF_6_ has an extremely long lifetime of 800–3200 years, so early SF_6_ reduction can result in substantial long-term climate benefits even beyond the current century. Similarly, PFCs also have very high GWPs and can be much more effectively reduced in Comprehensive GHG abatement.

While non-CO_2_ abatement measures effectively reduce cooling and industrial process emissions, agriculture emissions are barely affected by these measures because the cost-effective mitigation options are comparatively limited at current estimation. Agriculture is the largest non-CO_2_ emission source, contributing to half of the total non-CO_2_ emissions throughout the century. A previous work^31^ suggested that the economic potential of mitigation measures in the livestock sector is less than 10% of what is technically possible. In addition, agriculture CH_4_ emissions mostly from enteric fermentation are projected to constitute an increasingly larger share of total anthropogenic CH_4_ emissions even under direct CH_4_ mitigation scenarios^32^. The residual emissions from these sources would ultimately limit the ability to further reducing non-CO_2_ emissions and achieving additional climate benefits.

Spatially, Comprehensive GHG abatement achieves greater non-CO_2_ reductions in 2050 across all regions, especially in China and the U.S. (Fig. 3d). Greater non-CO_2_ reductions are also achieved in growing economies such as Brazil, South Asia, and Western Africa, where industrial process and cooling demands are projected to increase rapidly (Fig. 3d). Compared with the Reference, HFCs are reduced by over 80% in 2050 for most regions under Comprehensive GHG abatement. In contrast, limited reductions could be achieved purely due to the demand reductions under CO_2_-driven GHG abatement (Supplementary Fig. 10). Notably, for all regions, CO_2_-driven GHG abatement results in significant additional SF_6_ emissions, while such side effects of greater electrification to replace fossil fuels can be turned entirely into net benefits with SF_6_ abatement measures under Comprehensive GHG abatement. In addition, non-CO_2_ abatement can also help reduce agriculture CH_4_ and N_2_O emissions, especially for the U.S., Central Asia, and Northern Africa, partially offsetting the increased agriculture emissions in these regions due to the high biomass energy production (Supplementary Fig. 7). All trends above are consistent in 2100 (Supplementary Fig. 11), with greater additional benefits achieved in Comprehensive GHG abatement.

## Discussion

Scenarios avoiding global warming >1.5 °C and 2 °C require the combined mitigation of CO_2_ and non-CO_2_ GHG emissions. Coupling the latest global non-CO_2_ emission projection and mitigation data with a state-of-the-art integrated assessment model, we find that non-CO_2_ emission reductions associated with decarbonization-driven fuel switching and specific abatement measures can significantly contribute to climate change mitigation and greatly help to ease the burden of CO_2_ mitigation toward 1.5 °C and 2 °C targets. In particular, CO_2_ emissions would have to reach net-zero by two decades earlier to achieve the same climate target without specific non-CO_2_ abatement measures disentangled from major decarbonization actions. While it is critical to reach net-zero CO_2_ to stabilize global temperature change, our results emphasize that underestimating the role of non-CO_2_ mitigation measures can lead to substantial bias in lower carbon budgets and premature net-zero CO_2_ commitment.

Our research highlights the importance of comprehensive GHG abatement that fully accounts for non-CO_2_ mitigation measures in conjunction with CO_2_ emissions in deep mitigation scenarios. We incorporate the updated marginal abatement cost (MAC) curves for all major non-CO_2_ GHG species across economic sectors and global regions, and our year-specific parameterization considers future technological innovation in mitigation measures. In addition to fuel-related CH_4_ and N_2_O reductions from fuel switching and demand reduction driven by decarbonization, our results highlight the significant mitigation potential of F-gases from industrial processes and cooling sectors, which can be cost-effectively achieved for all global regions. Furthermore, our results also identify major sources of residual non-CO_2_ emissions that call for additional technological innovation and structural changes to reduce, following in line with the previous literature^21,33,34^. For example, agriculture non-CO_2_ emissions are barely affected under mitigation scenarios for most regions because of the relatively limited economic mitigation options available at current cost estimates. Without disruptive technological innovation that can significantly expand the affordable mitigation potential or leverage economic feedback of directly pricing CH_4_ that can lead to lifestyle changes^21^, it will be challenging to reduce non-CO_2_ emissions from agriculture. Future research in better understanding the technical and economic potential for all GHGs and technological/institutional innovation in further mitigating residual non-CO_2_ emissions can further contribute to the growing body of literature supporting the transformation needed to achieve ambitious climate stabilization.

The following sources of uncertainty should be noted. First, our 90 mitigation scenarios are examined within the same modeling framework sharing the same socioeconomic pathway, economic structure, and climate modeling assumptions, so the effect of non-CO_2_ emission reductions can be isolated. To examine the robustness of our findings, we further conducted two sets of sensitivity analyses to demonstrate the impact of alternative socioeconomic pathways and alternative technological change assumptions for non-CO_2_ abatement (Supplementary Figs. 2-3). For the main 1.5 °C scenario, socioeconomic and technological change assumptions can influence the ultimate level of non-CO_2_ emissions by up to −34% (SSP1) and +44% (no technological change), respectively.

Second, alternative assumptions in modeling structure, non-CO_2_ mitigation parameterization, and climate modeling choices that characterize the physical effects of climate forcers, can certainly affect the ultimate temperature changes^21,32,35^. Although other climate forcers such as black carbon, aerosols, and ozone, can also contribute to temperature change^36^ and interact with CO_2_ and non-CO_2_ GHG forcings through complex atmospheric processes^5,37^, their emissions are primarily affected by the level of CO_2_ mitigation^24^ instead of specific non-CO_2_ GHG mitigation measures. Third, while our climate results are unaffected by the GWP choices (Methods), the GWP assumptions can affect the relative contributions of different GHG species to the total GHG reductions when presenting. If a longer GWP timeframe were chosen, the long-lived CO_2_ emissions would show a greater share of the cumulative GHG reductions in terms of CO_2_-equivalent amount (Supplementary Figs. 4–6). Lastly, uncertainty in emission factors, derived from historical emissions, may influence the relative mitigation potential from some sectors and major emitting regions (Fig. 3). For example, several studies suggested a considerable underestimation of U.S. methane emission from oil and gas supply chain in some inventory data compared to ground-based, facility-scale measurements^38,39^, due to challenges in quantifying abnormal operating conditions, leakages, and emerging gas production techniques. Similarly, existing inventories provide divergent estimates in China’s coal mining methane emissions^40,41^, as methane emissions are not necessarily directly related to coal production but can also occur even if coal production is ceased^42^. Given that fossil fuel production is a major source of CH_4_ emissions, future work would benefit from more accurate estimation methodologies that better account for operational details and geologic factors of fossil fuel production. Nevertheless, key qualitative insights still hold: Comprehensive GHG abatement that fully incorporates non-CO_2_ mitigation measures in addition to a net-zero CO_2_ commitment facilitates a more moderate decline in emissions on the pathway to Paris stabilization goals.

The CO_2_ mitigation in our study is modeled with an economy-wide carbon price, and increased societal carbon prices also drive non-CO_2_ abatement through MAC curves (see Methods). Here we do not attempt to address the question of how likely a uniform, economy-wide carbon price would be applied in the real world^43^, but rather focus on to which degree the expected emission changes in all GHGs would collectively shape climate consequences. However, it should be noted that CO_2_ abatement often requires large technological shifts and demand reductions, which typically need high mitigation costs^44–47^. Conversely, many non-CO_2_ abatement technologies reflected in the MACs do not require radical changes in technologies from a user standpoint, making them potentially easier to widely adopt if appropriate incentives and information are provided. Thus, future work should seek to better understand the barriers and potential of both CO_2_ and non-CO_2_ reductions to devise more realistic mitigation scenarios to inform scientific modeling and decision-making. With increasing numbers of major economies announcing or indicating ambitious net-zero targets to pursue the path of net-zero emissions targets under the Paris Agreement, it is critical to understand the mitigation potential and specific abatement actions for all greenhouse gases.

## Methods

### Non-CO_2_ GHG data

Our emission data are based on the Community Emissions Data System (CEDS v_2020-09-11), which provides annual historical (1750–2019) anthropogenic chemically reactive gases (CO, CH_4_, NH_3_, NO_x_, SO_2_, NMVOCs), carbonaceous aerosols (black carbon—BC, and organic carbon—OC), and CO_2_. First published in ref. ^48^, the latest CEDS (DOI: 0.5281/zenodo.4025316) is updated with more recent inventory data, updated emission factors in selective sectors, and updated country-level emission inventories.

For non-CO_2_ GHGs (CH_4_, N_2_O, HFCs, PFCs, and SF_6_), we further harmonize our current CEDS emissions with the 2019 EPA Global Non-CO_2_ Greenhouse Gas Emission Projections and Mitigation Potential report^10^. This report provides country-, sector-, and year-specific emissions projections and estimates of mitigation potential for non-CO_2_ GHGs through a comprehensive global analysis using a combination of country-reported inventory data supplemented with EPA-estimated calculations consistent with inventory guidelines of the Intergovernmental Panel on Climate Change (IPCC)^27^. Historical emission estimates were incorporated from country-reported data from 1990 to 2015, and emissions were projected through 2050. Here we scaled our non-CO_2_ emissions to EPA historical emissions by region, sector, and species from 1990 to 2015, derived emission factors using historical activity data, and then projected future emissions being consistent with EPA’s emission projections.

The detailed non-CO_2_ GHG mitigation estimates in EPA report^10^ employ a bottom-up, engineering cost approach that analyzed the costs of a wide range of mitigation technologies and incorporated them into an economic estimate of MAC curve (Supplementary Fig. 12). MAC curves were estimated by the break-even price at which the present-value benefits and costs for each mitigation option equilibrate. Mitigation potential and cost also account for country differences in industry structures and availability, labor, nonenergy materials, and energy across countries. In addition, MACs incorporate the effects of technology change on mitigation costs, representing cost savings of mitigation measures due to technology learning over time.

For each region and species, mitigation cost and corresponding emission reductions achievable are summarized into source categories covering all sectors (Supplementary Tables 3–5). For example, CH_4_ mitigation includes control measures in resource production (coal mining activities, natural gas and oil system, and combustion of fossil fuels and biomass), agriculture (livestock and rice cultivation), and waste management (landfill of solid waste and wastewater). While HFC mitigation mainly happens in industrial processes and cooling sectors, such as electronics manufacturing, the use of substitutes for ozone-depleting substances, and HCFC-22 production. Major abatement measures for each source category are listed in Supplementary Table 6.

### The Global Change Analysis Model

GCAM (jgcri.github.io/gcam-doc/) is a multi-sector integrated assessment model that links the global economy, energy supply and demand, agriculture, land use, water, and climate systems^25^. GCAM is designed to explore long-term interactions between human and earth systems, which has been widely used to produce global emission scenarios, including the IPCC Special Report on Emissions Scenarios^49^, the Representative Concentration Pathways^50^, and quantification of the Shared Socioeconomic Pathways^51^. GCAM 5.3 used in this analysis includes 32 geo-political regions, and linked land allocation, water use, and agriculture production across over 300 subregions and 235 water basins.

GCAM simulates the evolution of the energy, land, water, climate, and economic systems driven by exogenous assumptions regarding population and labor productivity, determining energy and service demands for each region and each sector. CO_2_ emissions are estimated by tracking the carbon content of various fuels embedded in energy flows. Historical emissions of other GHGs (CH_4_ and N_2_O), short-lived forcing agents (BC and OC), and air pollutants (CO, SO_2_, NO_x_, and PM_2.5_) are adopted from the latest CEDS, which is then used to develop emission factors (emission per energy input or service output of a specific technology). Future emissions are estimated as the product of the projected economic activity and the corresponding emission factor for a given technology.

In this study, we harmonize historical emissions with the EPA non-CO_2_ report so that future non-CO_2_ emission trends are consistent with the baseline scenario in the EPA report. In addition, we incorporate MAC curves for non-CO_2_ emissions, mapped from the EPA mitigation scenario (Supplementary Note). Based on the maximum reduction potentials reported from 2015 to 2050 (Supplementary Table 7), we derived year-specific technological change ($TC$) parameter for 2020–2050, allowing GCAM to simulate the EPA MAC in these future years and to extend the current MAC after 2050 (Eq. 1). In the main analysis, we assume the ${TC}$ after 2050 is the average ${TC}$ for pre-2050 periods. Supplementary Fig. 12 qualitatively illustrates the effect of technological change on MACs.

1 $$E\left({t}_{1},p\right)=E\left({t}_{0},p\right)\times \mathop{\prod }\limits_{i={t}_{0}}^{{t}_{1}}(1+{{TC}}^{i})$$

Where $E\left({t}_{1},p\right)$ and $E\left({t}_{0},p\right)$ represent the emission reductions corresponding to mitigation cost of $p$ in the MAC of year ${t}_{1}$ and ${t}_{0}$, ${{TC}}^{i}$ represents the technology change in year $i$ relative to year ${t}_{0}$. Therefore, any future year’s mitigation potential is cumulatively determined by the ${TC}$s of all previous years. To avoid unrealistically abrupt emission reductions in the first several modeling periods due to the implementation of MAC (which does not necessarily reflect the delays in stock turnover), we phase in the maximum reduction potential from 2020, linearly reaching their reported maximum mitigation potential by 2040. In the sensitivity analysis, we further examine the effect of different ${TC}$ assumptions, which do not substantively alter our findings.

These MACs allow non-CO_2_ GHG emissions to respond to increased carbon prices (Supplementary Fig. 13) by considering the application of various control measures in addition to fuel switching and adopting more efficient technologies, based on both technological feasibility and cost-effectiveness reflected in MACs. For example, due to the limited economic mitigation measures in agriculture, CH_4_ emission is much more challenging to reduce relative to resource extraction. In GCAM, both MAC and direct pricing can be applied to non-CO_2_ GHG emissions. For our analysis, we assume that only the MAC affects non-CO_2_ emissions, reflecting the difficulties in transferring the emission pricing on non-CO_2_ GHG emissions to the final goods. If considering direct pricing, these emission penalties will likely be transferred to the corresponding economic activity and consumers, leading to economic feedbacks on technology shifts and behavioral changes^21^.

GCAM is coupled with Hector v2.5 (https://github.com/JGCRI/hector), an open-source, reduced-form global climate model^52,53^, to estimated various climate outcomes such as radiative forcings and global temperature. At every GCAM modeling period, GCAM supplies Hector with global emissions of fossil fuel and industrial CO_2_, LULUC, CH_4_, N_2_O, BC, OC, CO, NMVOC, and halocarbons. Next, emissions are interpolated into yearly estimations in Hector to calculate the corresponding future concentrations of GHGs from the input emissions. Then Hector calculates global mean radiative forcing from GHG concentrations and short-lived climate forcers and finally converts the radiative forcing to global mean temperature^52^. It should be noted that GWP-100 is only used to aggregated GHG emissions in our results, while our climate results (Fig. 1) are unaffected by GWP choices.

### Scenarios

In this study, we model a Reference scenario and 90 mitigation scenarios pairing 30 CO_2_ abatement pathways with three non-CO_2_ abatement levels. The Reference scenario is harmonized with the baseline scenario in the EPA mitigation report, assuming projected emission rates consistent with historical levels without future effects of policy changes beyond GCAM’s final calibration year (2015). However, future emissions are determined by both emission rates and projected activity. The Reference scenario in the current work projected slightly higher non-CO_2_ emissions compared with EPA’s baseline scenario, mainly from agriculture CH_4_ emissions driven by rapid growth in agricultural demand in developing countries. Reference also includes a small portion of non-CO_2_ GHG reductions at no cost that are readily available without abatement costs (Supplementary Table 8). Reference and all mitigation scenarios share the same socioeconomic growth trajectory based on a middle-of-the-road socioeconomic pathway (SSP2)^54^. Regional non-CO_2_ GHG emissions in the Reference scenario are shown in Supplementary Tables 9–15.

The CO_2_ abatement pathways are characterized by two levels of annual CO_2_ emissions as endpoints (0 and −8 GtCO_2_ yr^−1^), representing different societal expectations on negative CO_2_ emissions necessary to achieve 2 °C and 1.5 °C targets, respectively. The 2 °C pathways linearly reach net-zero CO_2_ emissions between 2030 and 2100 in 5-year increments, followed by zero CO_2_ emissions afterwards, and the 1.5 °C pathways linearly reach −8 GtCO_2_ yr^−1^ CO_2_ emissions between 2030 and 2100 in 5-year increments (so their net-zero CO_2_ years are slightly earlier), followed by −8 GtCO_2_ yr^−1^ CO_2_ emissions afterwards. The endpoints of 0 and −8 GtCO_2_ yr^−1^ CO_2_ are generally on the conservative side of negative CO_2_ emissions required for the corresponding 2 °C and 1.5 °C pathways in existing literature^26^.

Each CO_2_ abatement pathway is further evaluated under three non-CO_2_ abatement levels. CO_2_ abatement only assumes the same non-CO_2_ emissions as the Reference scenario while reducing CO_2_ emissions alone. This is a counterfactual scenario aiming at isolating the climate impact of CO_2_ mitigation alone. CO_2_-driven GHG abatement includes CO_2_ abatement and the non-CO_2_ abatement associated with fuel switching and demand reduction driven by CO_2_ abatement without explicit non-CO_2_ abatement measures. Finally, Comprehensive GHG abatement includes specific non-CO_2_ abatement measures, such as repairing leaks in existing large cooling systems to reduce HFC emissions and methane reductions from natural gas and oil systems with flaring. This is achieved by fully integrating country, sector, and year-specific MAC data from the EPA report into GCAM’s non-CO_2_ module, allowing non-CO_2_ emissions to respond to increased GHG prices by explicitly considering various control measures based on their technical feasibility and cost-effectiveness. The implementation of MACs allows us to fully evaluate the mitigation potential of non-CO_2_ emissions and its contribution to climate stabilization toward 1.5 °C.

## Supplementary information

Supplementary Information File

## Data Availability

The Community Emissions Data System (CEDS) data are publicly available at https://github.com/JGCRI/CEDS/wiki/Release-Notes. EPA non-CO_2_ mitigation data are publicly available at https://www.epa.gov/global-mitigation-non-co2-greenhouse-gases/global-non-co2-greenhouse-gas-emission-projections. The GCAM model output data generated in this study have been deposited in 10.5281/zenodo.5167496. Source data are provided with this paper.

## Source data

Source Data

## Code availability

GCAM is an open-source community model available at https://github.com/JGCRI/gcam-core/releases. The processing code associated with this study is available at 10.5281/zenodo.5165580.
