# Supplementary material for: Deep mitigation of CO2 and non-CO2 greenhouse gases toward 1.5 °C and 2 °C futures
Source: Nat Commun. 2021 Oct 29;12:6245. doi: 10.1038/s41467-021-26509-z (PMC8556229; doi:10.1038/s41467-021-26509-z)
Supplement: Supplementary file 1 — Supplementary Information File [file 41467_2021_26509_MOESM1_ESM.pdf]

Deep Mitigation of CO_2 and non-CO_2 Greenhouse Gases towards 1.5°C and 2°C Futures

Yang Ou[1], Christopher Roney[1§], Jameel Alsalam[2], Katherine Calvin[1], Jared Creason[2], Jae Edmonds[1], Allen A. Fawcett[2], Page Kyle[1], Kanishka Narayan[1], Patrick O'Rourke[1], Pralit Patel[1], Shaun Ragnauth[2], Steven J. Smith[1], Haewon McJeon[1*]

[1] Joint Global Change Research Institute, Pacific Northwest National Laboratory

[2] U.S. Environmental Protection Agency

[§] Present address: Electric Power Research Institute, 3420 Hillview Avenue, Palo Alto, CA, 94304, USA.

*Corresponding author: Haewon.McJeon@pnnl.gov

Supplementary Note: Incorporating EPA non-CO_2 GHG emission and mitigation data into GCAM

The 2019 EPA non-CO_2 GHG mitigation report[1] provides county, sector, and year-specific emissions projections and estimates of mitigation potential for non-CO_2 GHGs through a comprehensive global analysis. This report combines country-reported inventory data with EPA-estimated calculations consistent with the Intergovernmental Panel on Climate Change (IPCC) inventory guidelines. Historical emission estimates were incorporated from 195 country-reported data from 1990 through 2015, and emissions were projected through 2050.

The abatement analysis for all non-CO_2 emissions from sources in the agriculture, energy, waste, and industrial process sectors is based on the 2013 EPA Global Mitigation of non-CO_2 GHGs Report[2] and improves upon prior and subsequent studies. The non-CO_2 mitigation analysis and MAC curves account for the differences in industry structure across countries where sectoral data are available. Additionally, the 2019 EPA report accounted for country/regional differences in mitigation price through a series of international cost indices (labor, nonenergy materials, energy) to create a more heterogeneous representation of emissions and mitigation costs and benefits across countries. Thus, the 2019 EPA analysis provides considerable detail at the sector and subsector levels and across countries.

Here we scaled our non-CO_2 emissions to EPA historical emissions by region, sector, and species from 1990 to 2015, and the projected future emissions are generally consistent with the EPA's emission projections. In this study, we first harmonize GCAM historical non-CO_2 GHG emissions with EPA baseline projections for 1990, 2010, 2005, 2010, and 2015 (Supplementary Table 1, Supplementary Table 2). Next, we develop marginal abatement cost (MAC) curves for each control category based on EPA mitigation data and then map them into GCAM sectors (Supplementary Tables 3-5). Supplementary Table 6 provides major mitigation measures under each source category[1].

Supplementary Table 7 summarizes MAC control categories and their maximum reduction potential by year. Some categories already have relatively high mitigation potential at the current level (in 2015 and 2020), such as Nitric and Adipic Acid Production; some categories start with a relatively low mitigation potential but increase substantially in the future, such as Aerosols Product Use. The different mitigation potential across sources and

their growth rates are comprehensively represented through MAC options in GCAM for various sources and modeling periods (through year-specific “technological changes” feature in MAC).

Supplementary Table 8 summarizes the average MAC reduction at no cost. In GCAM, zero-cost MAC reduction is always applied even without any carbon price. This portion of emission reduction represents mitigation efforts at no cost, therefore the emission projection in the GCAM Reference scenario already includes these reductions. Furthermore, these zero-cost reductions will be endogenously increased driven by the underlying technological change assumptions.

Supplementary Figures

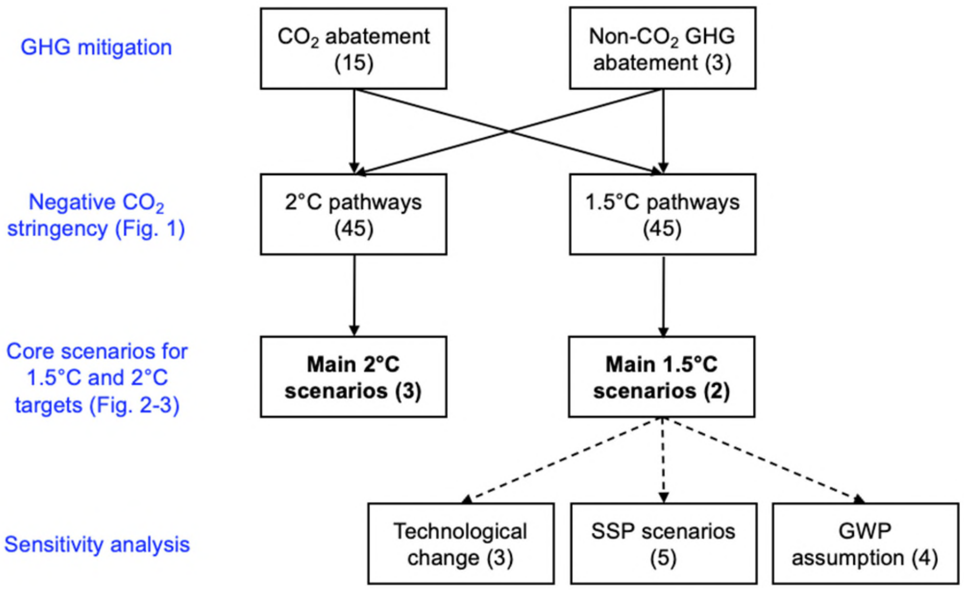

Supplementary Figure 1 Analytical framework. Numbers in parentheses represent the number of scenarios modeled in this study. "CO_2 abatement" represents different CO_2 mitigation pathways reaching net-0 $GtCO_2$ yr^{-1} (for 2°C pathways) or reaching net -8 $GtCO_2$ yr^{-1} (for 1.5°C pathways), respectively. "Non-CO_2 GHG abatement" represents different levels of non-CO_2 GHG abatement measures (Methods). "1.5°C pathways" and "2°C pathways" are terms used to characterize different levels of net negative CO_2 emissions (0 or -8 $GtCO_2$ yr^{-1}) to identify the main 1.5°C and 2°C scenarios, while not all 1.5°C and 2°C pathways can reach 1.5°C and 2°C stabilizations.

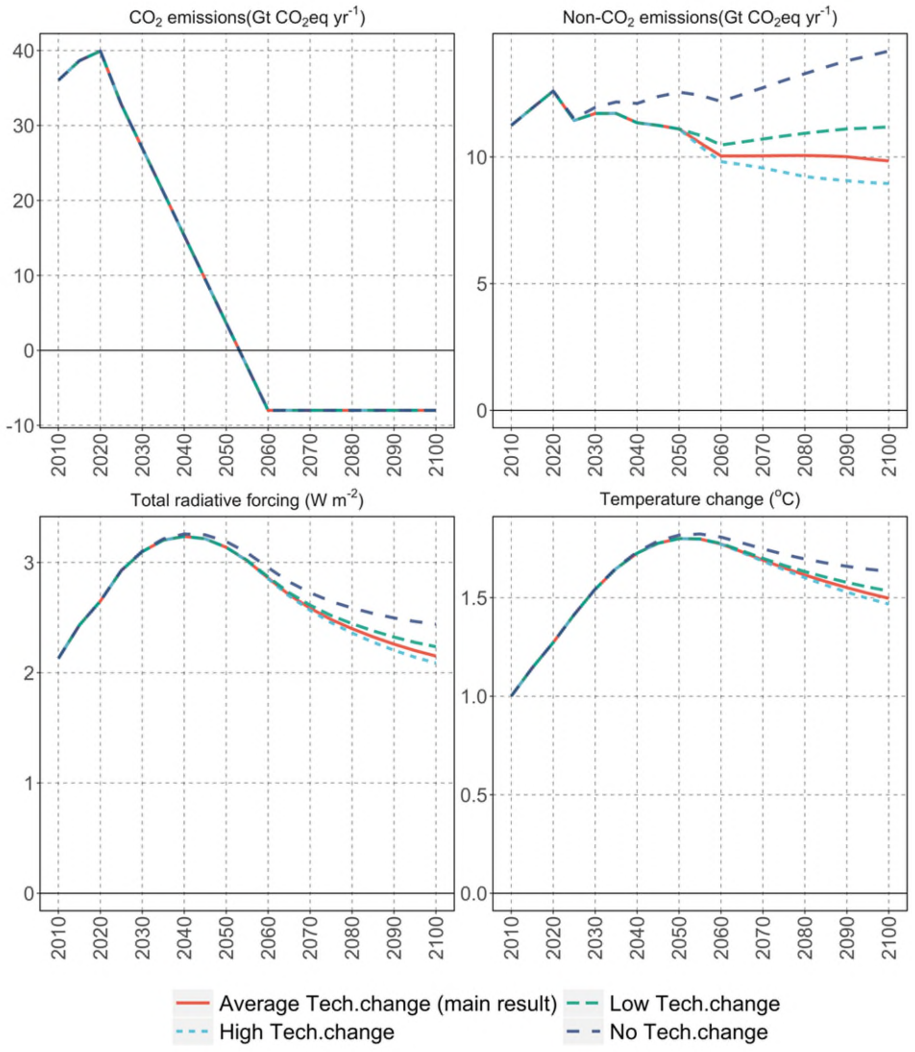

Supplementary Figure 2 Emission and climate results of comprehensive GHG mitigation scenario when reaching net-zero CO_2 emissions by 2053 (and -8 $GtCO_2$ yr^{-1} by 2060) under alternative assumptions of technology change after 2050. "Average Tech.change" assumes post-2050 technological change of MACs as the average technology change from 2020 to 2050; "Low Tech.change" assumes post-2050 technological changes of MACs are zeros; "High Tech.change" assumes post-2050 technological change of MACs after 2050 as the highest technology change among years of 2020-2050; "No Tech.change" assumes no technological change effect for MACs for all modeling periods. Non-CO_2 GHG emissions were aggregated with GWP-100 from ref[3].

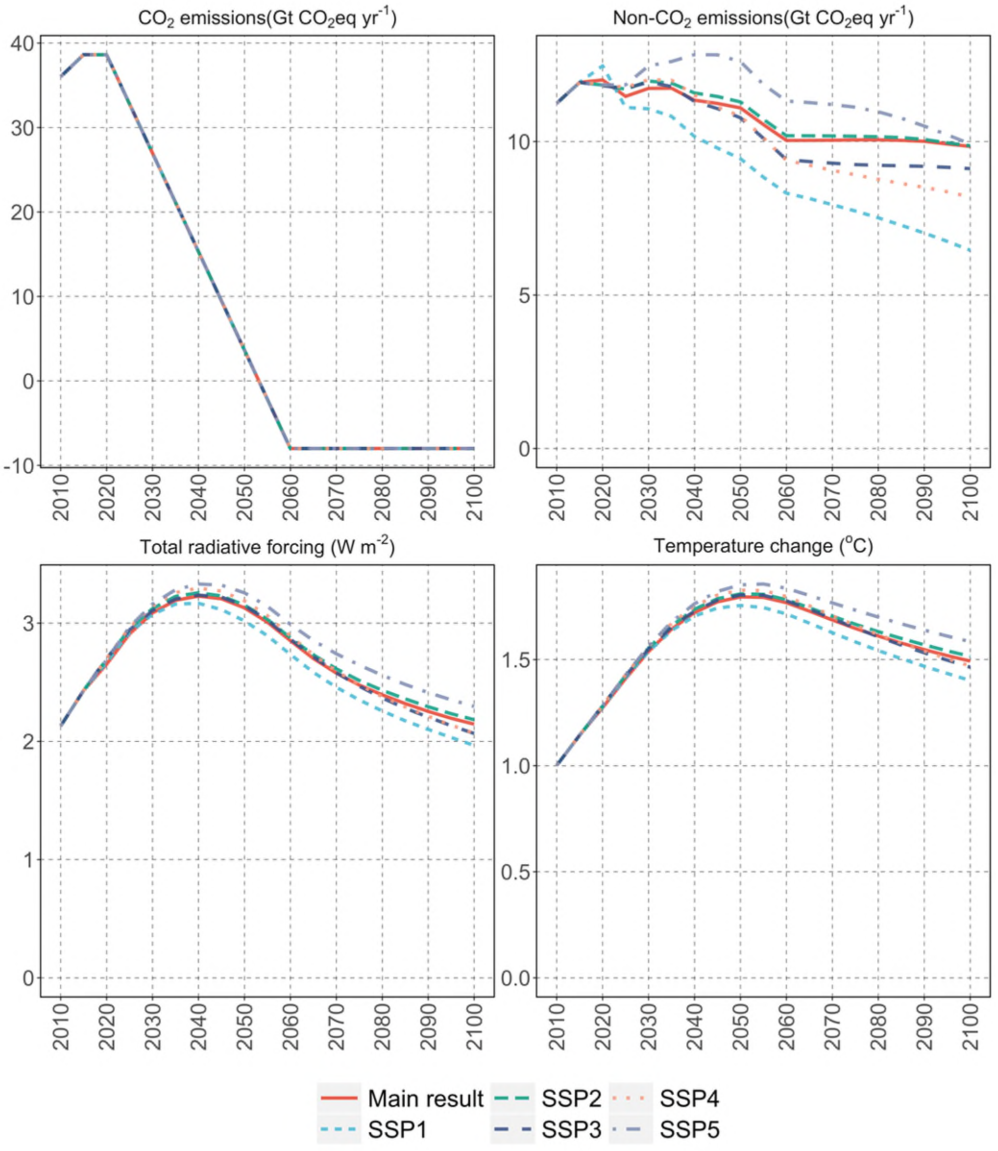

Supplementary Figure 3 Emission and climate results of comprehensive GHG mitigation scenario when reaching net-zero CO_2 emissions by 2053 (and -8 $GtCO_2$ yr^{-1} by 2060) across five SSP scenarios. The GCAM *Reference* scenario shares the same socioeconomic assumptions (population and GPD per capita assumptions) with the SSP2 pathway, which drives the overall energy demand. However, there are some differences between the main scenarios and SSP2 for other assumptions, such as industrial energy use, cement production, agriculture, CCS supply, and resource availability. These differences lead to a very small difference in nonCO_2 emissions shown in this figure. Non-CO_2 GHG emissions were aggregated with GWP-100 from ref[3]. Detailed GCAM modeling assumptions for SSP scenarios can be found in the public release package (https://github.com/JGCRI/gcam-core).

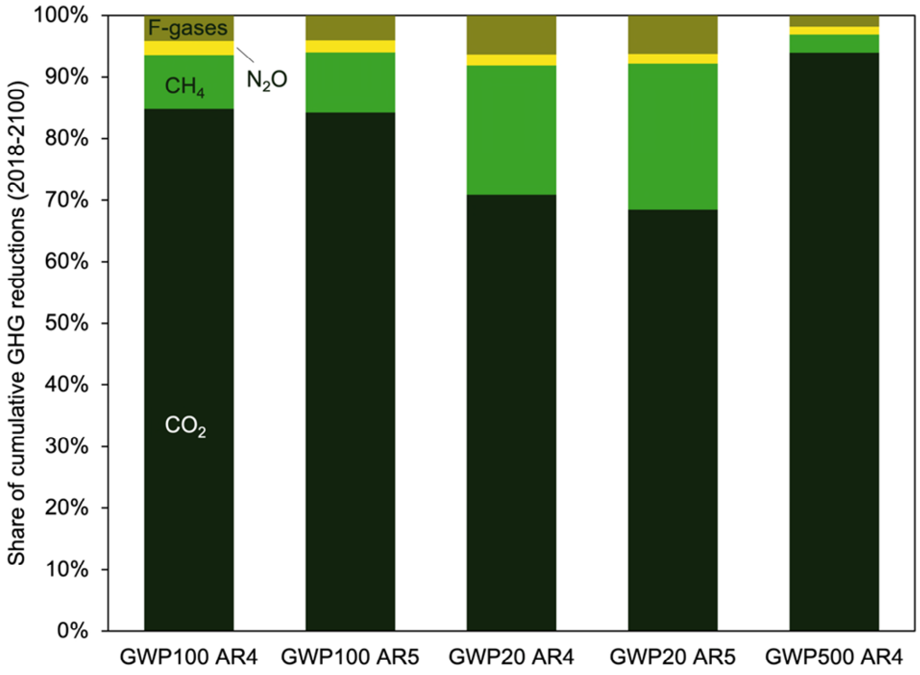

Supplementary Figure 4 Share of cumulative GHG reductions (2018-2100) in the Comprehensive GHG Mitigation scenario when reaching net-zero CO_2 emissions by 2053 (and -8 $GtCO_2$ yr^{-1} by 2060) across five Global Warming Potentials (GWPs) assumptions.

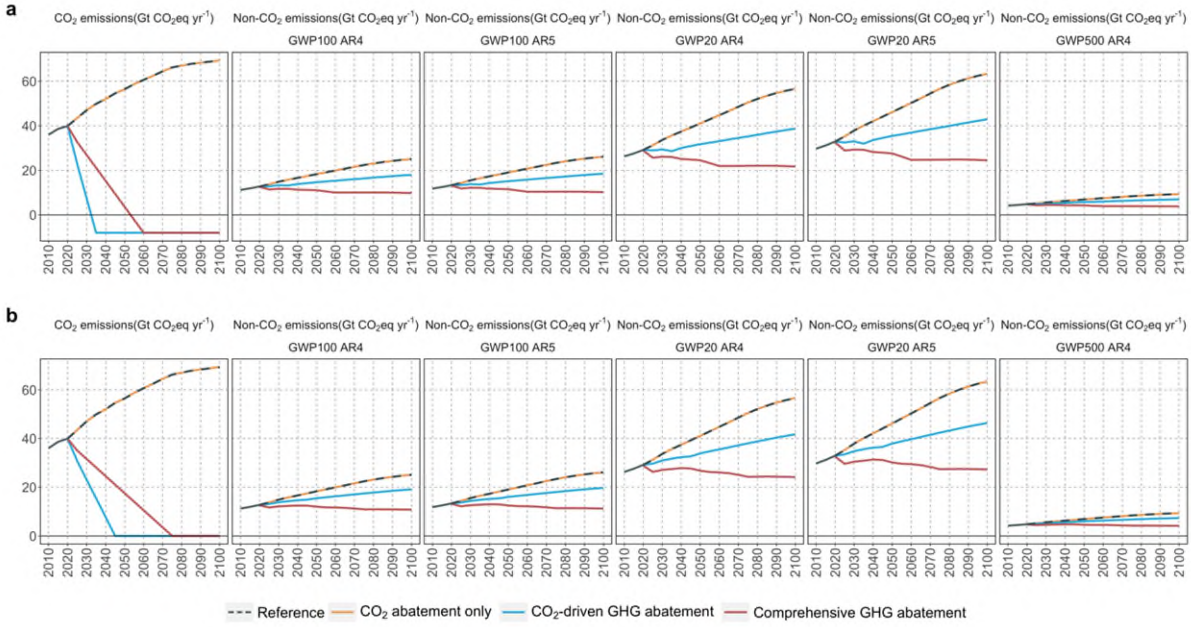

Supplementary Figure 5 Global CO_2 and non-CO_2 GHG emission results highlighting (a) 1.5°C and (b) 2°C scenarios. Non-CO_2 GHG emissions were aggregated with five GWP assumptions.

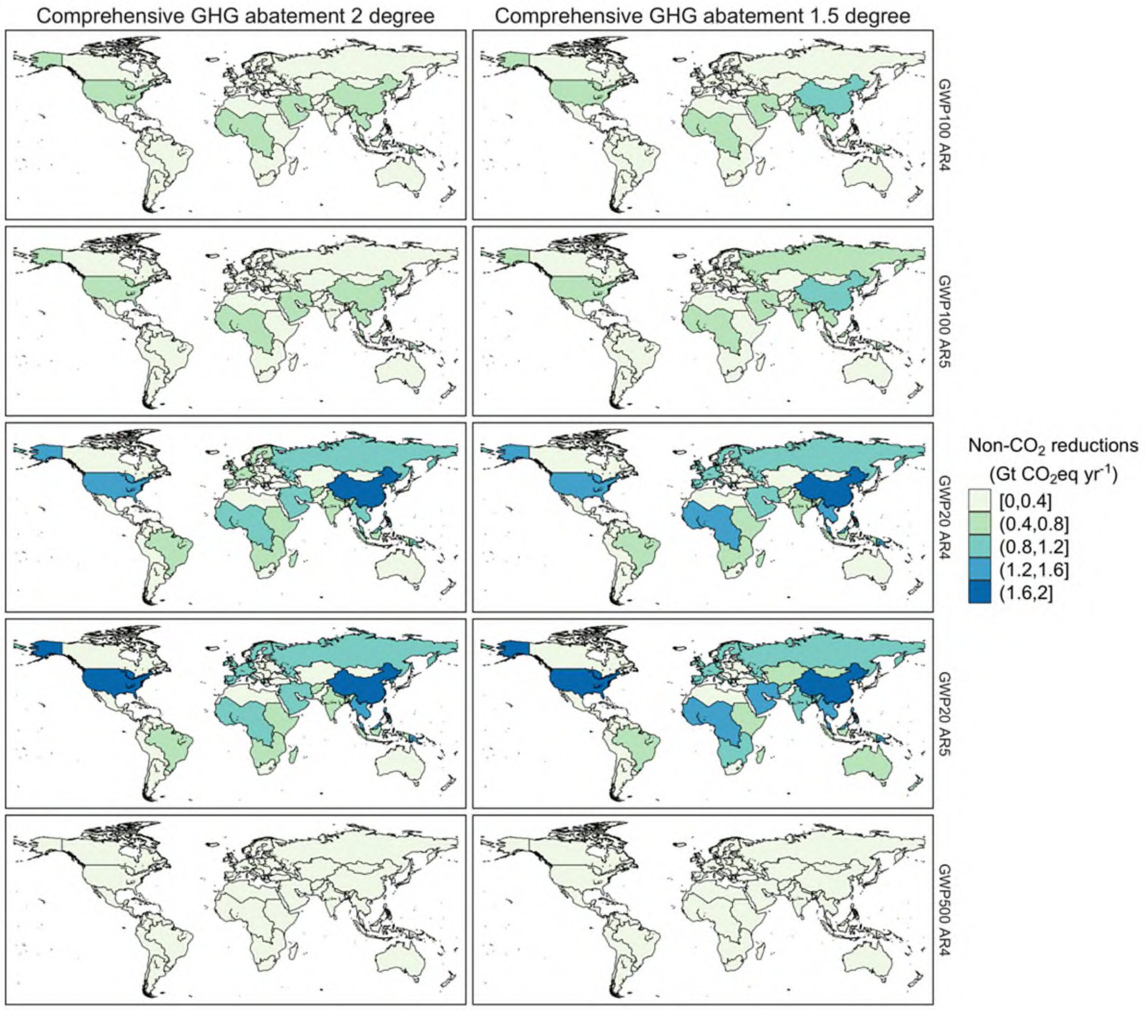

Supplementary Figure 6 Non-CO_2 GHG reductions in 2050 by GCAM regions. Non-CO_2 GHG emissions were aggregated with five GWP assumptions. "Comprehensive GHG abatement 2 degree" scenario reaches net-zero CO_2 emissions by 2075, and "Comprehensive GHG abatement 1.5 degree" scenario reaches net-zero CO_2 emissions by 2053 (and -8 $GtCO_2$ in 2060). Global maps in this figure are created using an open-source R package[4] and documented in ref[5].

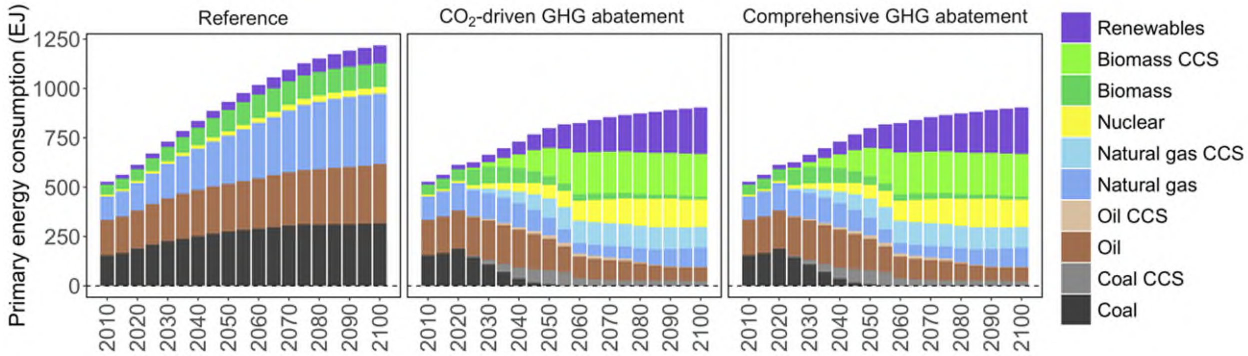

Supplementary Figure 7 Primary energy consumption in Reference, and CO_2-driven GHG abatement and Comprehensive GHG abatement mitigation scenarios when reaching net-zero CO_2 by 2053 (and -8 $GtCO_2$ yr^{-1} by 2060). As CO_2 emissions are reduced to net negative levels since 2055, fossil energy is substantially displaced by renewables and biomass CCS. CO_2 targets are assumed to be constant at the 2060 level (-8 $GtCO_2$ yr^{-1}) afterward, so the post-2060 energy transition is smoother than in the pre-2060 periods.

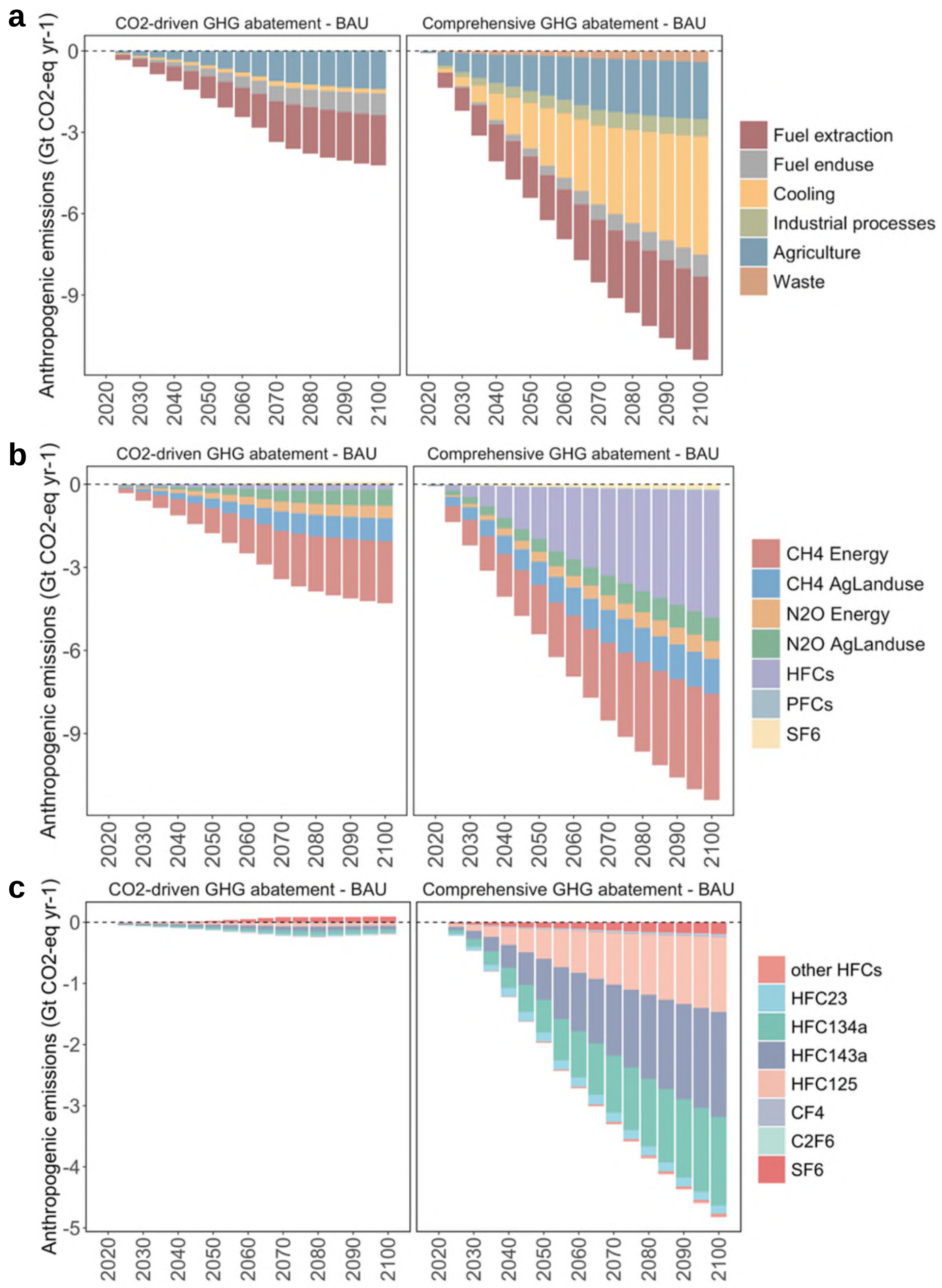

Supplementary Figure 8 Changes of non-CO_2 emission by sector (a), species (b), and changes of F-gas by species (c) relative to the Reference when reaching net-zero CO_2 by 2053 (and -8 Gt CO_2 yr^{-1} by 2060). Non-CO_2 GHG emissions were aggregated with GWP-100 from ref[3].

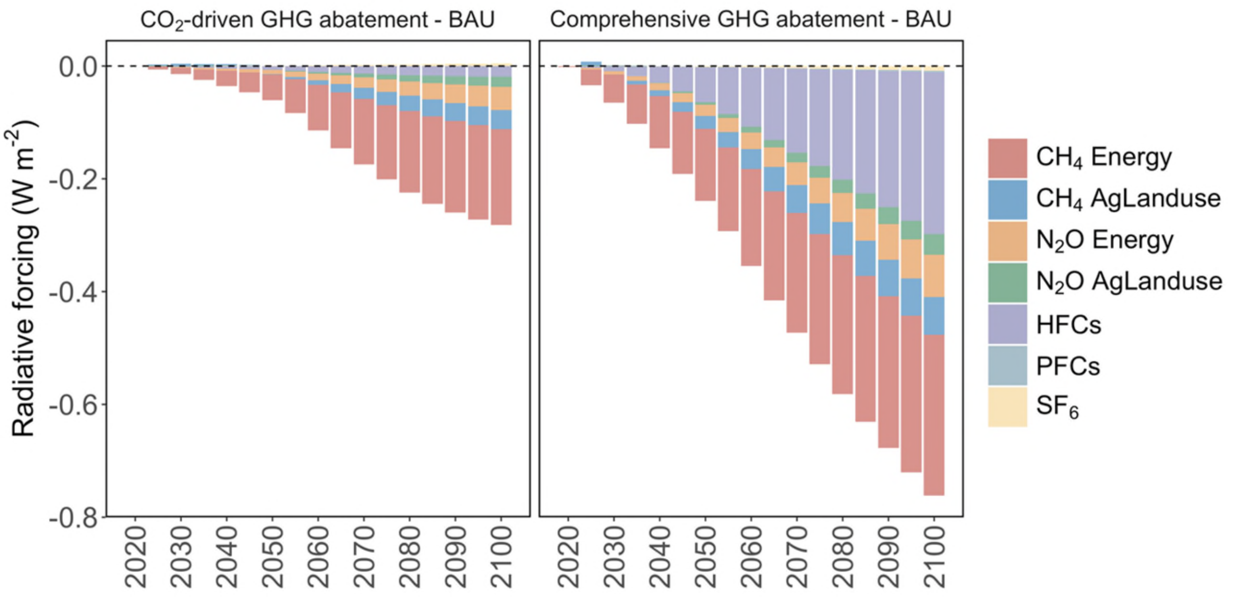

Supplementary Figure 9 Changes of non-CO_2 GHG forcing by species relative to the Reference when reaching net-zero CO_2 by 2053 (and -8 Gt CO_2 yr^{-1} by 2060).

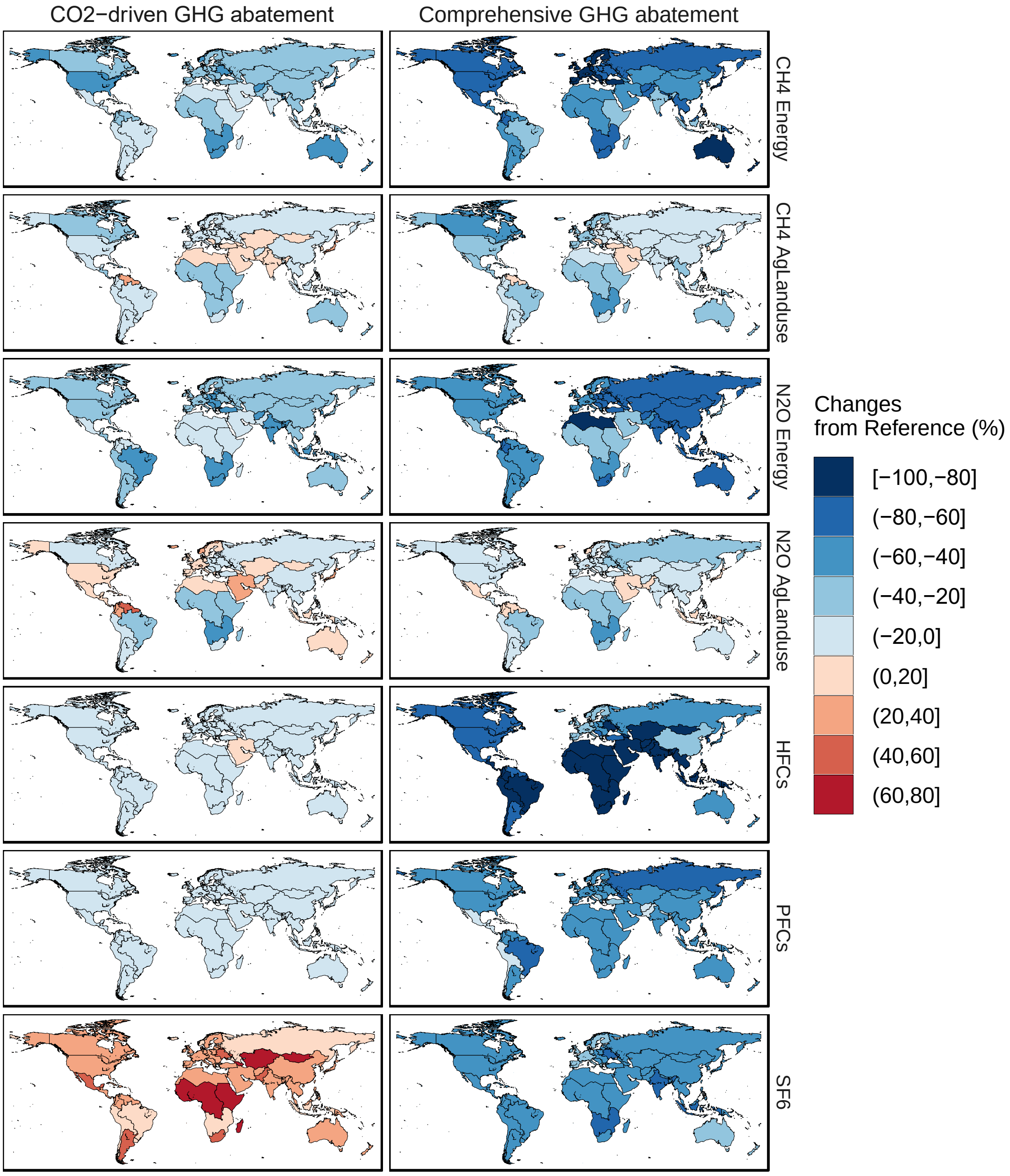

Supplementary Figure 10 Regional non-CO_2 GHG emission reductions relative to Reference in 2050 when reaching net-zero CO_2 emissions by 2053 (and -8 $GtCO_2$ yr^{-1} in 2060). Here the Comprehensive GHG abatement limits temperature change below 1.5°C in 2100. Global maps in this figure are created using an open-source R package[4] and documented in ref[5].

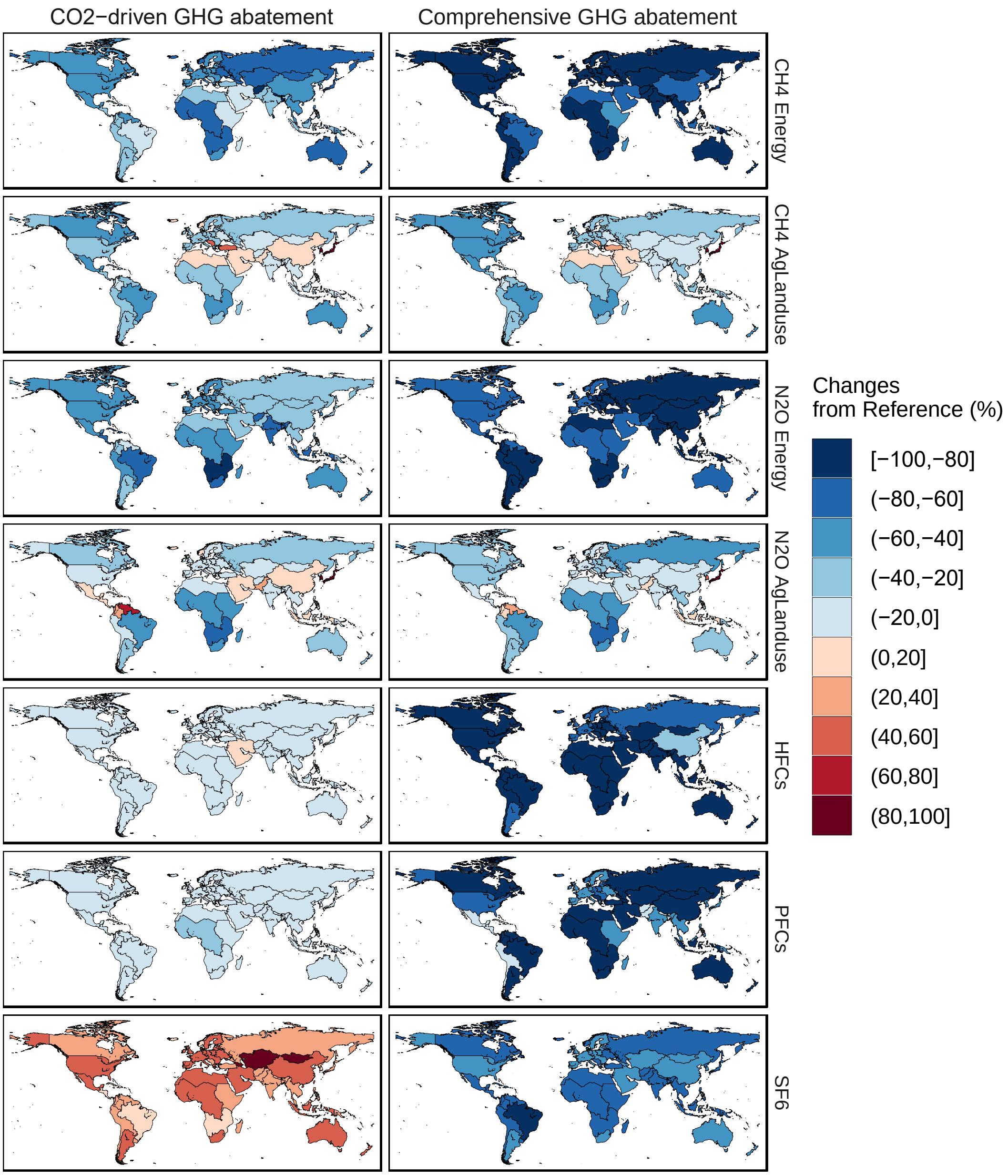

Supplementary Figure 11 Regional non-CO_2 GHG emission reductions relative to Reference in 2100 when reaching net-zero CO_2 emissions by 2053 (and -8 $GtCO_2$ yr^{-1} in 2060). Here the Comprehensive GHG abatement limits temperature change below 1.5°C in 2100. Global maps in this figure are created using an open-source R package[4] and documented in ref[5].

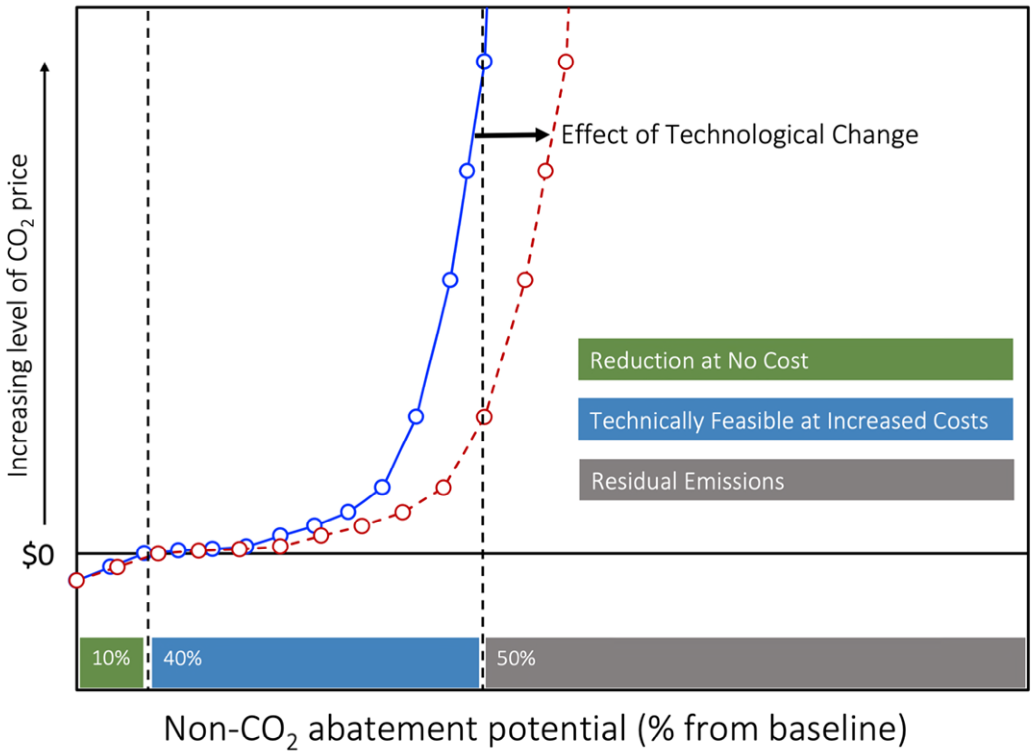

Supplementary Figure 12 Illustrative marginal abatement cost (MAC) curve.

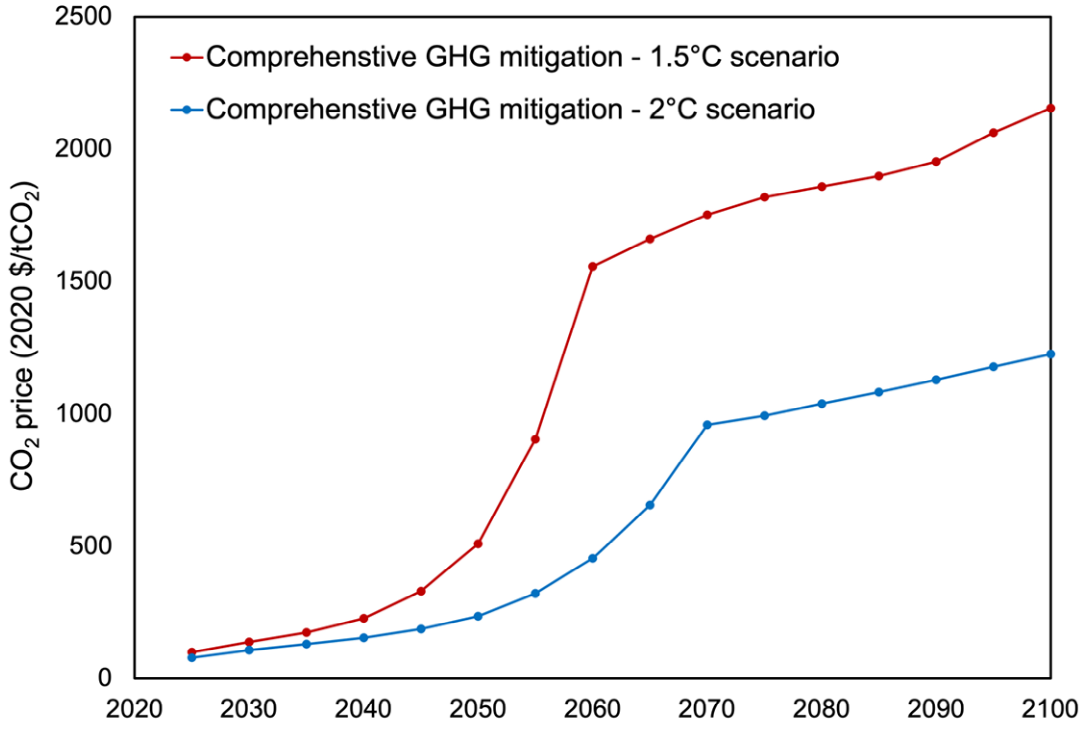

Supplementary Figure 13 CO_2 prices (2020$ /tCO_2) for core scenarios. In "Comprehensive GHG mitigation - 1.5°C" scenario, CO_2 emissions linearly reduce to -8 $GtCO_2$ yr^{-1} in 2060 and stay constant. In "Comprehensive GHG mitigation - 2°C" scenario, CO_2 emissions linearly reduce to 0 $GtCO_2$ yr^{-1} in 2075 and stay constant.

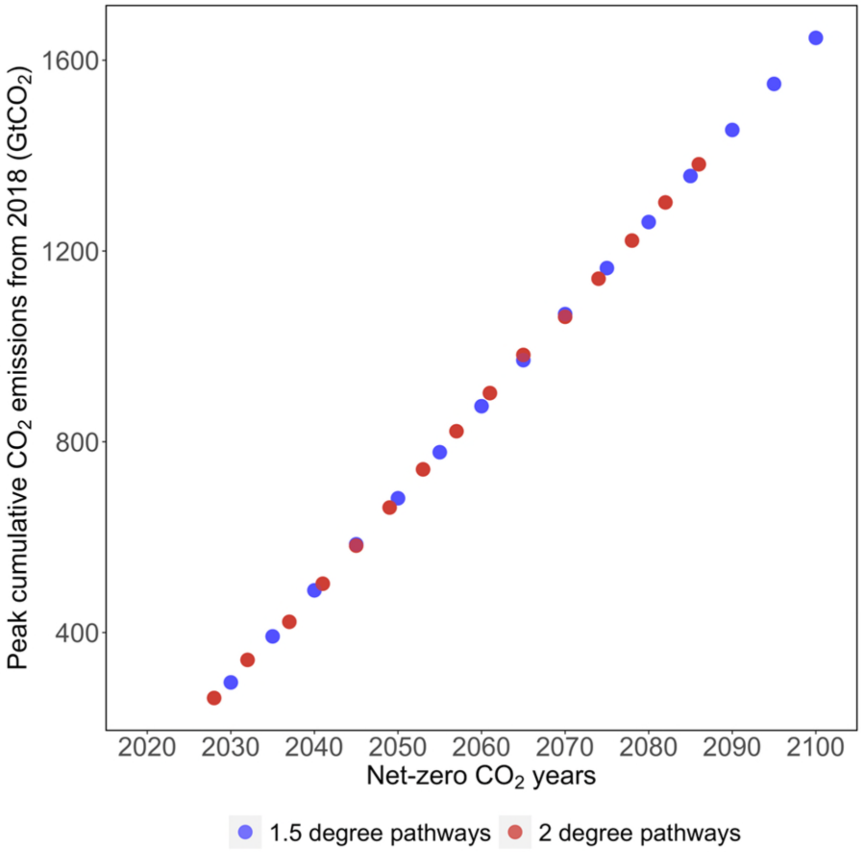

Supplementary Figure 14 Linear relationship between net-zero years and the remaining carbon budget. “1.5°C pathways” and “2°C pathways” assume CO_2 emissions linearly reduce to -8 and 0 $GtCO_2$ yr^{-1} and stay constant, correspondingly.

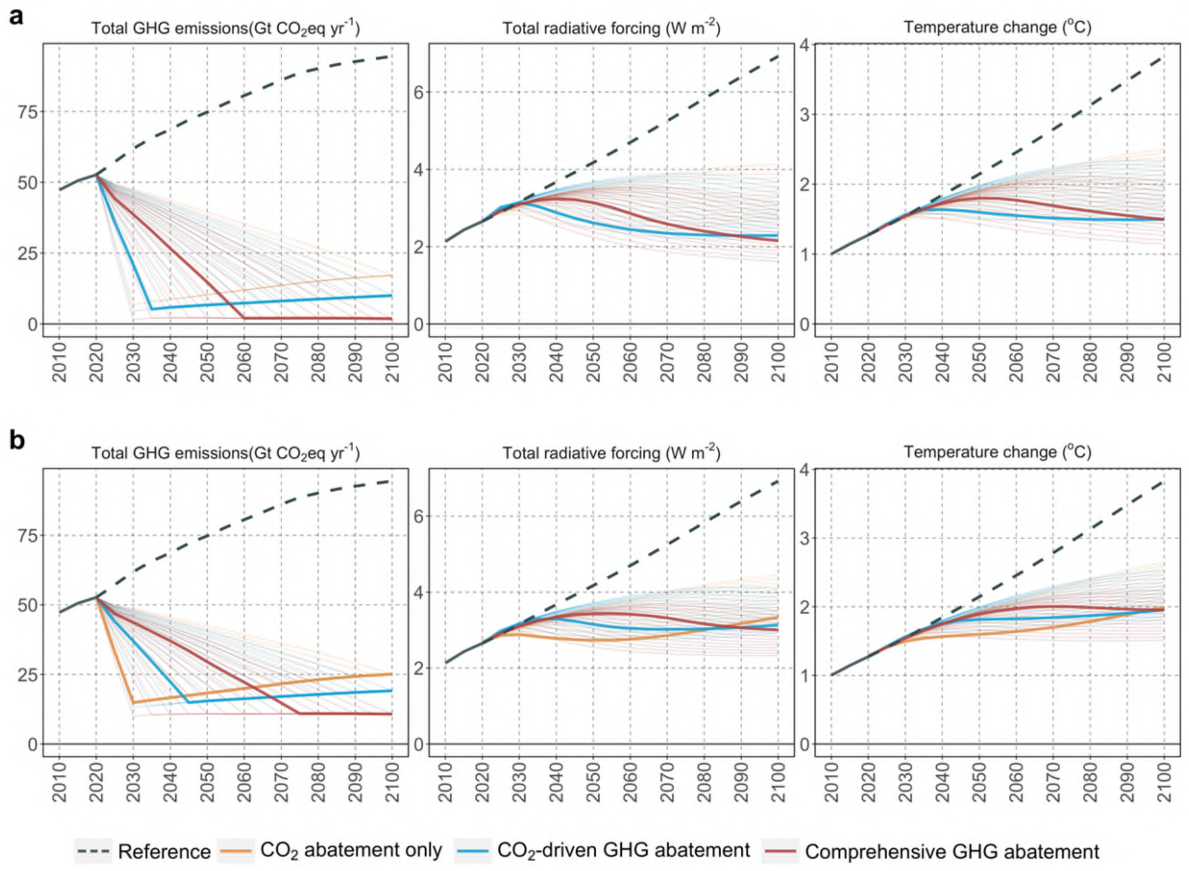

Supplementary Figure 15 Global emission and climate results highlighting (a) 1.5°C and (b) 2°C scenarios, including total GHG emissions, total radiative forcing, and global mean surface temperature change. "*Reference*" assumes no GHG mitigation. "*CO_2 abatement only*" cannot achieve the 1.5°C target under all modeled 1.5°C pathways but achieves the 2°C target if reaching net-zero CO_2 by 2030 under 2°C pathways; "*CO_2-driven GHG abatement*" achieves the 1.5°C target if reaching net-zero CO_2 by 2032 under 1.5°C pathways or achieves the 2°C target if reaching net-zero CO_2 by 2045 under 2°C pathways; "*Comprehensive GHG abatement*" achieves the 1.5°C target if reaching net-zero CO_2 by 2053 under 1.5°C pathways or achieves the 2°C target if reaching net-zero CO_2 by 2075 under 2°C pathways. The faint lines are colored based on non-CO_2 mitigation pathways, representing scenarios with CO_2 emission constraints reaching net-zero in alternative years. Non-CO_2 GHG emissions were aggregated with GWP-100 from ref[3].

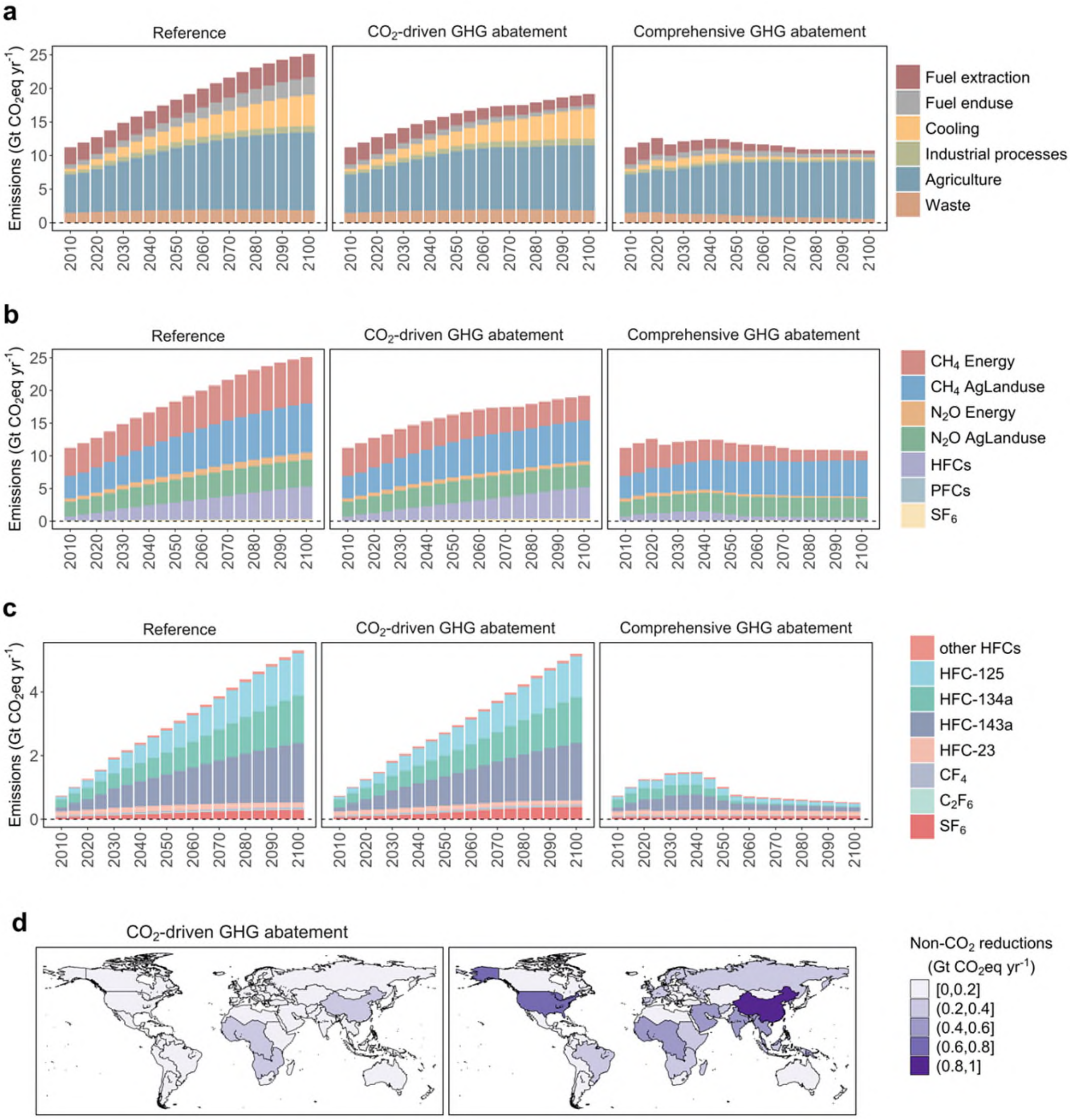

Supplementary Figure 16 Global non-CO_2 GHG emissions by sector (a) and species (b), F-gas emissions by species (c), and reductions in 2050 (d), when reaching net-zero CO_2 emissions by 2075 (and keep consistent) for a 2°C scenario. “other HFCs” include HFC-32, HFC-43, HFC-152a, HFC-227ea, HFC-236fa, HFC-245fa, and HFC-365mfc. Here the Comprehensive GHG mitigation scenario meets the 2°C targets without net negative CO_2 emissions. Global maps in this figure are created using an open-source R package[4] and documented in ref[5].

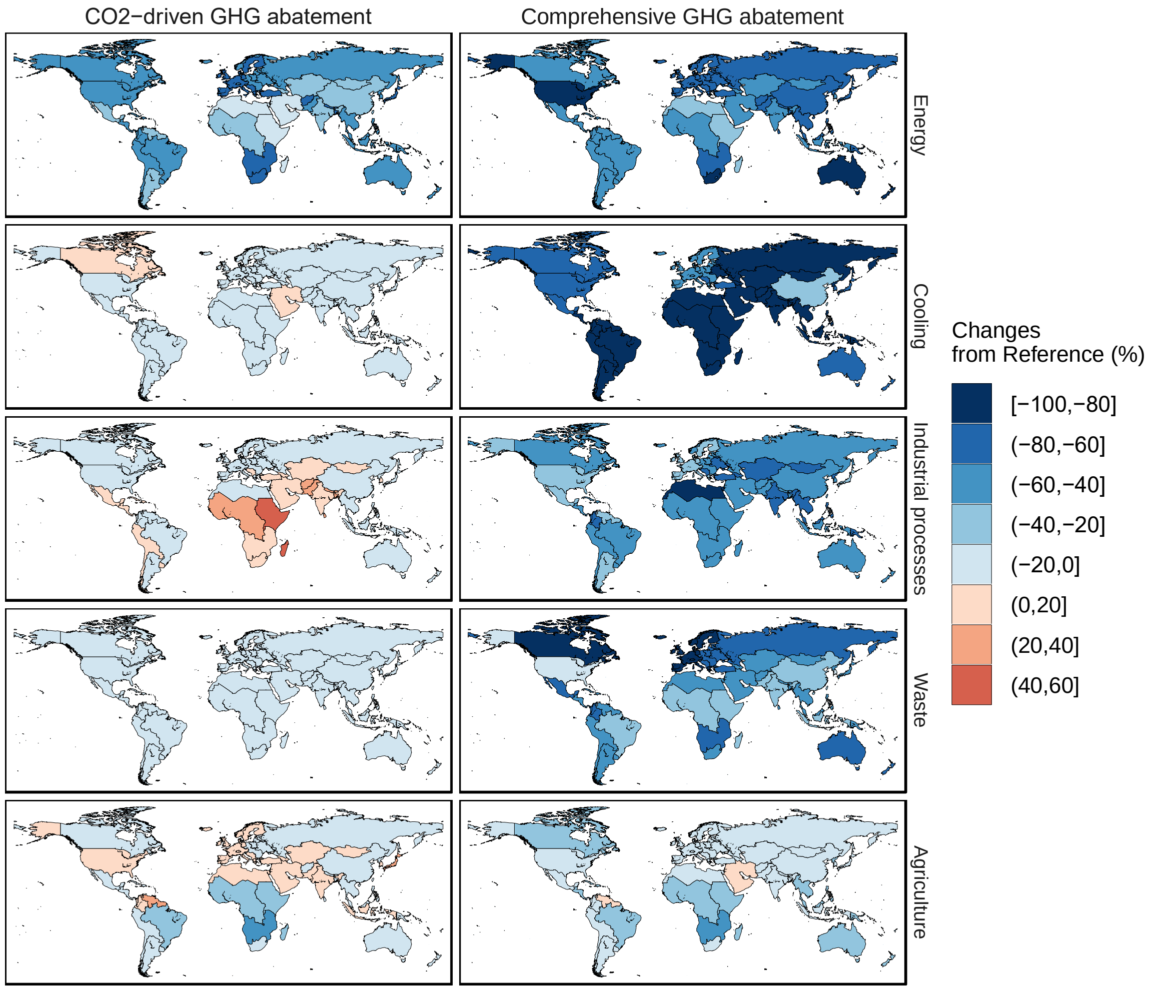

Supplementary Figure 17 Regional non-CO_2 GHG emission reductions relative to Reference in 2050 by sector when reaching net-zero CO_2 emissions by 2053 (and -8 $GtCO_2$ yr^{-1} in 2060). Here the Comprehensive GHG abatement limits temperature change below 1.5°C in 2100. Global maps in this figure are created using an open-source R package[4] and documented in ref[5].

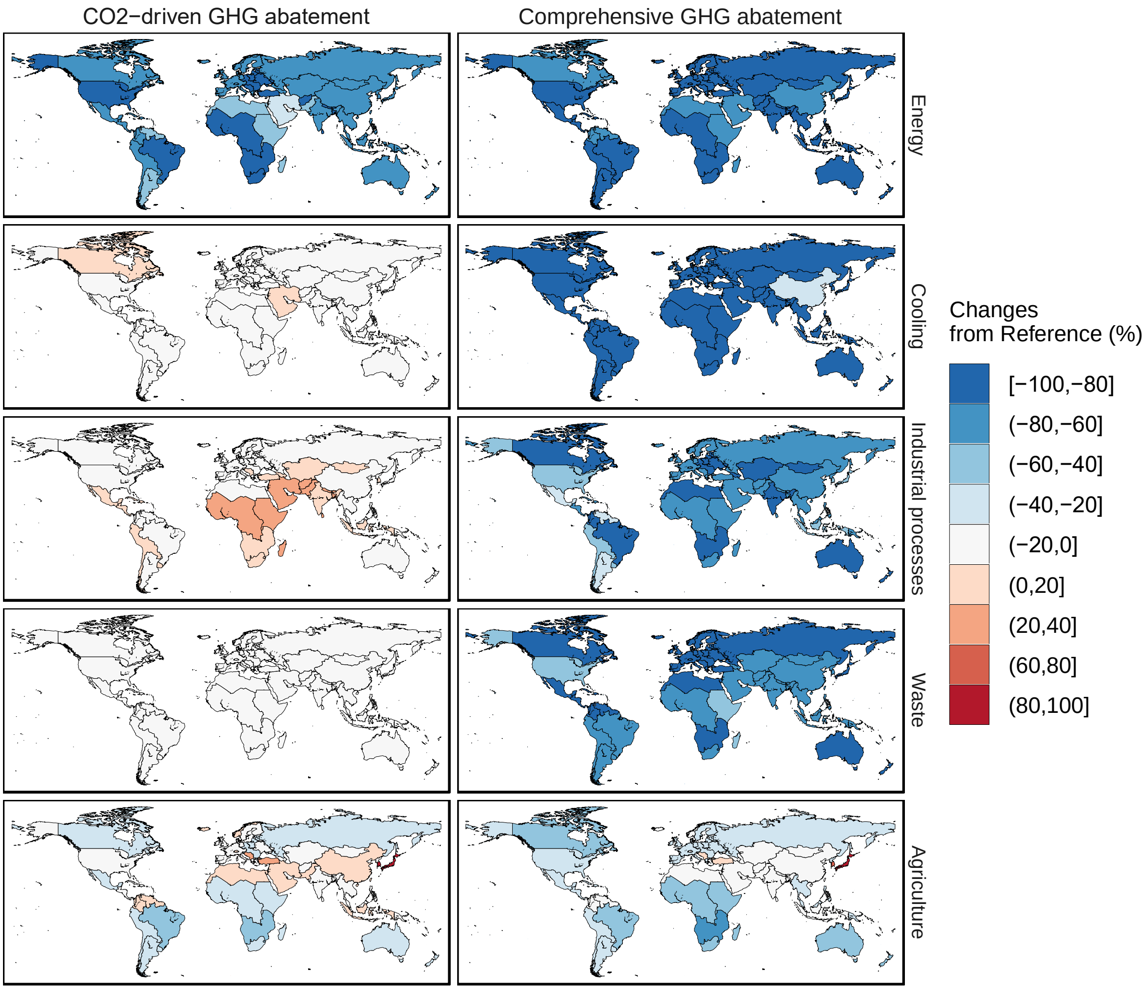

Supplementary Figure 18 Regional non-CO_2 GHG emission reductions relative to Reference in 2100 by sector when reaching net-zero CO_2 emissions by 2053 (and -8 $GtCO_2$ yr^{-1} in 2060). Here the Comprehensive GHG abatement limits temperature change below 1.5°C in 2100. Global maps in this figure are created using an open-source R package[4] and documented in ref[5].

Supplementary Tables

Supplementary Table 1 GHG modeled in GCAM and their global warming potential (GWP-100). GWP-100 in AR4[3] is used to convert non-CO_2 GHGs into CO_2-equivalent (CO_2-eq).

GHG category	GHG	GWP-100 AR4	GWP-100 AR5	GWP-20 AR4	GWP-20 AR5	GWP-500 AR4
CO_2	CO_2	1	1	1	1	1
CH_4	CH_4	25	28	72	84	7.6
N_2O	N_2O	298	265	289	264	153
HFCs	HFC23	14,800	12,400	12,000	10,800	12,200
	HFC32	675	677	2,330	2,430	205
	HFC125	3,500	3,170	6,350	6,090	1,100
	HFC134a	1,430	1,300	3,830	3,710	435
	HFC143a	4,470	4,800	5,890	6,940	1,590
	HFC152a	124	138	437	506	38
	HFC227ea	3,220	3,350	5,310	5,360	1,040
	HFC43	1,640	1,650	4,140	4,310	500
	HFC236fa	9,810	8,060	8,100	6,940	7,660
	HFC365mfc	794	804	2,520	2,660	241
	HFC245fa	1,030	858	3,380	2,920	314
PFCs	CF_4	7,390	6,630	5,210	4,880	11,200
	C_2F_6	12,200	11,100	8,630	8,210	18,200
SF6	SF6	22,800	23,500	16,300	17,500	32,600

Supplementary Table 2 Sectoral coverage of non-CO_2 GHG in EPA non-CO_2 mitigation report[1] and included in the current study.

**Sector[1]**	**IPCC Category**	**CH_4**	**NO_2**	**HFCs**	**PFCs**	**SF_6**
Energy						
Natural Gas and Oil Systems	1B2 Oil and Natural Gas	X				
Coal Mining Activities	1B1 Solid Fuels	X				
Fuel Combustion						
Stationary and Mobile Combustion	1A Fuel Combustion Activities	X	X			
Biomass Combustion	1A Fuel Combustion (Biomass)	X	X			
Other Energy Sources	1B1 Solid Fuels		X			
	1B2 Oil and Natural Gas		X			
	4C Incineration and Open Burning of Waste	X	X			
Industrial processes						
Nitric and Adipic Acid Production						
Nitric Acid Production	2B2 Nitric Acid Production		X			
Adipic Acid Production	2B3 Adipic Acid Production		X			
Uses of Substitutes for Ozone-Depleting Substances	2F1 Refrigeration and Air Conditioning			X		
	2F2 Foam Blowing Agents			X		
	2F3 Fire Protection			X		
	2F4 Aerosols			X		
	2F5 Solvents			X		
	2F6 Other Applications			X		
HCFC-22 Production	2B9a By-product Emissions			X		
Electric Power Systems	2G1 Electrical Equipment					X
Metals Production						
Primary Aluminum Production	2C3 Aluminum Production				X	
Magnesium Production	2C4 Magnesium Production					X
Electronics Manufacturing	2E1 Semiconductor			X	X	X
	2E2 TFT Flat Panel Display			X	X	X
	2E3 Photovoltaics			X	X	X
Other Industrial Processes Sources	2A Mineral Industry	X	X			
	2B Chemical Industry	X				
	2C Metal Industry	X				
Agriculture						
Croplands (non-rice Croplands)	3C4 and 3C5 Direct/Indirect Emissions from Managed Soils		X			
Livestock Management						
Enteric Fermentation	3A1 Enteric Fermentation	X				
Manure Management	3A2 and 3C6 Manure Management	X	X			
Rice Cultivation	3C7 Rice Cultivations	X				
Other Agriculture Sources	3C4 and 3C5 Managed Soils	X	X			
	3C1 Emissions from Biomass Burning	X	X			
	3D Other	X				
Waste						
Landfills	4A Solid Waste Disposal	X				
Wastewater	4D Wastewater Treatment and Discharge	X	X			
Other Waste Sources	4E Other	X	X			

Supplementary Table 3 Sectoral mapping of GCAM technologies into EPA category for energy-related CH_4 and N_2O.

**GCAM supplysector**	**GCAM subsector**	**GCAM technology**	**EPA category**
district heat	biomass	biomass	Combustion_Bio
electricity	biomass	biomass (conv)	Combustion_Bio
industrial energy use	biomass	biomass	Combustion_Bio
process heat cement	biomass	biomass	Combustion_Bio
resid heating	biomass	biomass	Combustion_Bio
resid others	biomass	biomass	Combustion_Bio
comm heating	biomass	biomass	Combustion_Bio
comm others	biomass	biomass	Combustion_Bio
resid heating	traditional biomass	traditional biomass	Combustion_Bio
resid others	traditional biomass	traditional biomass	Combustion_Bio
district heat	coal	coal	Combustion_general
district heat	gas	gas	Combustion_general
district heat	refined liquids	refined liquids	Combustion_general
electricity	coal	coal (conv pul)	Combustion_general
electricity	gas	gas (CC)	Combustion_general
electricity	gas	gas (steam/CT)	Combustion_general
electricity	refined liquids	refined liquids (steam/CT)	Combustion_general
industrial energy use	coal	coal	Combustion_general
industrial energy use	gas	gas	Combustion_general
industrial energy use	refined liquids	refined liquids	Combustion_general
N fertilizer	coal	coal	Combustion_general
N fertilizer	gas	gas	Combustion_general
N fertilizer	refined liquids	refined liquids	Combustion_general
process heat cement	coal	coal	Combustion_general
process heat cement	gas	gas	Combustion_general
process heat cement	refined liquids	refined liquids	Combustion_general
resid cooling	gas	gas	Combustion_general
resid heating	coal	coal	Combustion_general
resid heating	gas	gas	Combustion_general
resid heating	refined liquids	refined liquids	Combustion_general
resid others	coal	coal	Combustion_general
resid others	gas	gas	Combustion_general
resid others	refined liquids	refined liquids	Combustion_general
trn_aviation_intl	International Aviation	Liquids	Combustion_general
trn_freight	Domestic Ship	Liquids	Combustion_general

trn_freight	Freight Rail	Coal	Combustion_general
trn_freight	Freight Rail	Liquids	Combustion_general
trn_freight_road	Truck (>12t)	Liquids	Combustion_general
trn_freight_road	Truck (>12t)	NG	Combustion_general
trn_freight_road	Truck (0-2.7t)	Liquids	Combustion_general
trn_freight_road	Truck (0-2.7t)	NG	Combustion_general
trn_freight_road	Truck (2.7-4.5t)	Liquids	Combustion_general
trn_freight_road	Truck (2.7-4.5t)	NG	Combustion_general
trn_freight_road	Truck (4.5-12t)	Liquids	Combustion_general
trn_freight_road	Truck (4.5-12t)	NG	Combustion_general
trn_pass	Domestic Aviation	Liquids	Combustion_general
trn_pass	Passenger Rail	Liquids	Combustion_general
trn_pass_road	Bus	Liquids	Combustion_general
trn_pass_road	Bus	NG	Combustion_general
trn_pass_road_LDV_2W	Motorcycle (>250cc)	Liquids	Combustion_general
trn_pass_road_LDV_4W	Compact Car	Liquids	Combustion_general
trn_pass_road_LDV_4W	Compact Car	NG	Combustion_general
trn_pass_road_LDV_4W	Large Car	Liquids	Combustion_general
trn_pass_road_LDV_4W	Large Car	NG	Combustion_general
trn_pass_road_LDV_4W	Light Truck and SUV	Liquids	Combustion_general
trn_pass_road_LDV_4W	Light Truck and SUV	NG	Combustion_general
trn_pass_road_LDV_4W	Midsize Car	Liquids	Combustion_general
trn_pass_road_LDV_4W	Midsize Car	NG	Combustion_general
trn_shipping_intl	International Ship	Liquids	Combustion_general
trn_freight_road	Truck (0-2t)	Liquids	Combustion_general
trn_freight_road	Truck (0-2t)	NG	Combustion_general
trn_freight_road	Truck (2-5t)	Liquids	Combustion_general
trn_freight_road	Truck (2-5t)	NG	Combustion_general
trn_freight_road	Truck (5-9t)	Liquids	Combustion_general
trn_freight_road	Truck (5-9t)	NG	Combustion_general
trn_freight_road	Truck (9-16t)	Liquids	Combustion_general
trn_freight_road	Truck (9-16t)	NG	Combustion_general
trn_pass_road_LDV	Three-Wheeler	Liquids	Combustion_general
trn_pass_road_LDV	Three-Wheeler	NG	Combustion_general
trn_pass_road_LDV_4W	Mini Car	Liquids	Combustion_general

trn_pass_road_LDV_4W	Mini Car	NG	Combustion_general
trn_pass_road_LDV_4W	Multipurpose Vehicle	Liquids	Combustion_general
trn_pass_road_LDV_4W	Multipurpose Vehicle	NG	Combustion_general
trn_pass_road_LDV_4W	Subcompact Car	Liquids	Combustion_general
trn_pass_road_LDV_4W	Subcompact Car	NG	Combustion_general
trn_freight_road	Truck (0-1t)	Liquids	Combustion_general
trn_freight_road	Truck (0-1t)	NG	Combustion_general
trn_freight_road	Truck (1-6t)	Liquids	Combustion_general
trn_freight_road	Truck (1-6t)	NG	Combustion_general
trn_freight_road	Truck (6-30t)	Liquids	Combustion_general
trn_freight_road	Truck (6-30t)	NG	Combustion_general
trn_freight_road	Truck (>15t)	Liquids	Combustion_general
trn_freight_road	Truck (>15t)	NG	Combustion_general
trn_freight_road	Truck (6-15t)	Liquids	Combustion_general
trn_freight_road	Truck (6-15t)	NG	Combustion_general
trn_pass_road_LDV_2W	Moped	Liquids	Combustion_general
trn_pass_road_LDV_4W	Large Car and SUV	Liquids	Combustion_general
trn_pass_road_LDV_4W	Large Car and SUV	NG	Combustion_general
trn_freight_road	Truck (0-4.5t)	Liquids	Combustion_general
trn_freight_road	Truck (0-4.5t)	NG	Combustion_general
trn_freight_road	Truck (4.5-15t)	Liquids	Combustion_general
trn_freight_road	Truck (4.5-15t)	NG	Combustion_general
trn_pass_road_LDV_4W	Van	Liquids	Combustion_general
trn_pass_road_LDV_4W	Van	NG	Combustion_general
trn_freight_road	Truck (>32t)	Liquids	Combustion_general
trn_freight_road	Truck (>32t)	NG	Combustion_general
trn_freight_road	Truck (0-3.5t)	Liquids	Combustion_general
trn_freight_road	Truck (0-3.5t)	NG	Combustion_general
trn_freight_road	Truck (16-32t)	Liquids	Combustion_general
trn_freight_road	Truck (16-32t)	NG	Combustion_general
trn_freight_road	Truck (3.5-16t)	Liquids	Combustion_general
trn_freight_road	Truck (3.5-16t)	NG	Combustion_general
trn_freight_road	3W Rural	Liquids	Combustion_general
trn_freight_road	3W Rural	NG	Combustion_general
trn_freight_road	Truck (>14t)	Liquids	Combustion_general

trn_freight_road	Truck (>14t)	NG	Combustion_general
trn_freight_road	Truck (0-6t)	Liquids	Combustion_general
trn_freight_road	Truck (0-6t)	NG	Combustion_general
trn_freight_road	Truck (6-14t)	Liquids	Combustion_general
trn_freight_road	Truck (6-14t)	NG	Combustion_general
trn_pass_road_bus	Heavy Bus	Liquids	Combustion_general
trn_pass_road_bus	Heavy Bus	NG	Combustion_general
trn_pass_road_bus	Light Bus	Liquids	Combustion_general
trn_pass_road_bus	Light Bus	NG	Combustion_general
trn_pass_road_LDV_2W	Scooter	Liquids	Combustion_general
comm cooling	gas	gas	Combustion_general
comm heating	coal	coal	Combustion_general
comm heating	gas	gas	Combustion_general
comm heating	refined liquids	refined liquids	Combustion_general
comm others	coal	coal	Combustion_general
comm others	gas	gas	Combustion_general
comm others	refined liquids	refined liquids	Combustion_general
trn_freight_road	Heavy truck	Liquids	Combustion_general
trn_freight_road	Heavy truck	NG	Combustion_general
trn_freight_road	Light truck	Liquids	Combustion_general
trn_freight_road	Light truck	NG	Combustion_general
trn_freight_road	Medium truck	Liquids	Combustion_general
trn_freight_road	Medium truck	NG	Combustion_general
trn_pass_road_LDV	2W and 3W	Liquids	Combustion_general
trn_pass_road_LDV_4W	Car	Liquids	Combustion_general
trn_pass_road_LDV_4W	Car	NG	Combustion_general
trn_pass_road_LDV_4W	Large Car and Truck	Liquids	Combustion_general
trn_pass_road_LDV_4W	Large Car and Truck	NG	Combustion_general
trn_pass_road_LDV	2W and 3W	NG	Combustion_general
out_resources	coal	coal	Coal Mining activity
out_resources	natural gas	natural gas	Natural gas production
out_resources	crude oil	crude oil	Oil production
industrial processes	adipic acid	adipic acid	Industrial processes Adipic
industrial processes	nitric acid	nitric acid	Industrial processes Nitric
industrial processes	HCFC_22_Prod	HCFC_22_Prod	Industrial processes Other
industrial processes	other industrial processes	other industrial processes	Industrial processes Other

industrial processes	solvents	solvents	Industrial processes Other
urban processes	landfills	landfills	Waste
urban processes	wastewater treatment	wastewater treatment	Waste
urban processes	waste_incineration	waste_incineration	Waste

Supplementary Table 4 Sectoral mapping of GCAM technologies into EPA category for F-gases in cooling and industrial processes.

GCAM supplysector	GCAM subsector	GCAM technology	EPA category
cooling	electricity	electricity	ODS Substitutes: Refrigeration / Air Conditioning
industrial processes	foams	foams	ODS Substitutes: Foam Blowing
industrial processes	solvents	solvents	ODS Substitutes: Solvents
urban processes	aerosols	aerosols	ODS Substitutes: MDI Aerosols
urban processes	fire_exting	fire_exting	ODS Substitutes: Fire Extinguishing
industrial processes	HCFC_22_Prod	HCFC_22_Prod	Production of HCFC-22
industrial processes	semiconductors	semiconductors	Manufacture of Semiconductors
industrial processes	Al_Mg	Al_Mg	Magnesium Manufacturing
industrial processes	semiconductors	semiconductors	Flat Panel Display Manufacturing
industrial processes	Al_Mg	Al_Mg	Aluminum
industrial processes	semiconductors	semiconductors	Photovoltaic Manufacturing
electricity_net_ownuse	electricity_net_ownuse	electricity_net_ownuse	Electric Power Systems

Supplementary Table 5 MAC control categories for each EPA emission sources

EPA sector	EPA source	EPA subsource	Process
Agriculture	AgSoils	NA	Cropland Management
Agriculture	Livestock	Enteric	Livestock
Agriculture	Livestock	Manure	Livestock
Agriculture	OtherAg	NA	Cropland Management
Agriculture	Rice	NA	Rice Cultivation
Energy	Combustion	Biomass	NA
Energy	Coal	Surface	Coal Mining Activities
Energy	Coal	Under	Coal Mining Activities
Energy	NGO	Gas Product	Natural Gas and Oil Systems
Energy	NGO	Gas TSD	Natural Gas and Oil Systems
Energy	NGO	Oil Product	Natural Gas and Oil Systems
Energy	NGO	Oil Refining	Natural Gas and Oil Systems
Energy	OtherEnergy	NA	NA
Energy	Combustion	StatMob	NA
Industrial Processes	Metals	Aluminum	Primary Aluminum Production
Industrial Processes	EPS	NA	Electric Power Systems
Industrial Processes	Electronics	FPD	Flat Panel Display Manufacturing
Industrial Processes	HCFC22	NA	HCFC-22 Production
Industrial Processes	Metals	Magnesium	Magnesium Manufacturing
Industrial Processes	NitricAdipic	Adipic	Nitric and Adipic Acid Production
Industrial Processes	NitricAdipic	Nitric	Nitric and Adipic Acid Production
Industrial Processes	ODSSubs	Aerosols	Aerosols Product Use
Industrial Processes	ODSSubs	Fire Ext	Fire Protection
Industrial Processes	ODSSubs	Foams	Foams
Industrial Processes	ODSSubs	RefAC	Refrigeration and Air Conditioning
Industrial Processes	ODSSubs	Solvents	Solvent Use
Industrial Processes	OtherIPPU	NA	NA
Industrial Processes	Electronics	PV	Photovoltaic Cell Manufacturing
Industrial Processes	Electronics	Semi	Semiconductor Manufacturing
Waste	Landfills	Industrial	Landfilling and Solid Waste
Waste	Landfills	MSW	Landfilling of Solid Waste
Waste	OtherWaste	NA	NA
Waste	Wastewater	Rural	Wastewater
Waste	Wastewater	Urban	Wastewater

Supplementary Table 6 Major abatement measures for each EPA emission sources[1]

EPA category	Major abatement measures
Coal Mining	- Ventilation air methane (VAM) Oxidation,<br>- Degasification for Power Generation
Natural Gas and Oil Systems	- Directed Inspection and Maintenance<br>- Installing Vapor Recovery Units on Oil Storage Tanks<br>- Flaring Instead of Venting on Shallow Water Platforms
Nitric and Adipic Acid Production	- Thermal Destruction<br>- Non-selective Catalytic Reduction<br>- Tail-gas Catalytic Decomposition
Electronics: Semiconductor, FPD, and PV Manufacturing	- Thermal Abatement<br>- NF_3 Remote Clean<br>- Catalytic Abatement
Electric Power Systems	- Improved SF_6 Handling<br>- SF_6 Recycling<br>- Equipment Refurbishment
Metals: Aluminum and Magnesium Production	- Minor Retrofit (process computer control systems only<br>- Major Retrofit (process computer control systems + alumina point feeding)<br>- Alternative Cover Gas - Novec™ 612
Substitutes for Ozone-Depleting Substances (ODS)	- Leak Repair for Existing Large Equipment<br>- Refrigerant Recovery at Disposal for Existing Refrigeration/AC Equipment<br>- NH_3 and CO_2 in Cold Storage and Industrial Process Refrigeration (IPR)
HCFC-22 Production	- Thermal Oxidation
Livestock Enteric Fermentation and Manure Management	- Antimethanogen<br>- Propionate Precursors<br>- Improved Feed Conversion
Croplands Emissions from Agricultural Soil Management	- Reduced Fertilizer<br>- No Till<br>- Nitrification Inhibitor Fertilizer
Rice Cultivation	- MD With Nitrification Inhibitor Fertilizer<br>- AWD With Nitrification Inhibitor<br>- No Till
Landfills	- Electricity Generation with a Reciprocating Engine<br>- Flaring of Landfill Gas<br>- Waste to Energy
Wastewater	- Latrine to Aerobic WWTP<br>- Open Sewer to Aerobic WWTP<br>- Septic Tank to Aerobic WWTP

Supplementary Table 7 Globally aggregated maximum MAC reduction levels for all controlling categories from 2015 to 2050. Categories are sorted by the ratio of their 2050 values to their 2015 values as an indicator of technological improvement of various mitigation measures.

EPA sector	EPA Source	GCAM MAC control	2015	2020	2025	2030	2035	2040	2045	2050	2050/2020
Industrial	FOAM	Foams	0.00%	0.04%	0.50%	1.21%	1.73%	1.58%	2.11%	3.33%	83.03
Industrial	FIRE	Fire Protection	0.00%	0.14%	0.59%	0.97%	1.64%	2.03%	2.26%	2.31%	16.62
Industrial	RAC	Refrigeration and Air Conditioning	0.00%	6.60%	28.53%	39.19%	51.10%	60.75%	76.81%	85.45%	12.94
Industrial	AERO	Aerosols Product Use	0.00%	11.63%	34.75%	48.21%	51.31%	52.25%	52.25%	52.25%	4.49
Industrial	SOLV	Solvent	0.00%	4.00%	6.46%	9.45%	12.15%	13.98%	14.10%	14.18%	3.54
Industrial	AL	Primary Aluminum Production	23.55%	27.19%	30.65%	33.95%	37.08%	40.06%	42.89%	45.58%	1.68
Waste	WWR	Wastewater	20.39%	25.34%	30.79%	36.72%	36.61%	36.50%	36.39%	36.29%	1.43
Energy	GAS	Natural Gas and Oil Systems	30.59%	33.43%	35.93%	38.32%	40.43%	42.46%	44.34%	46.12%	1.38
Industrial	HCFC	HCFC-22 Production	31.35%	81.87%	85.89%	88.17%	90.74%	92.55%	93.81%	94.71%	1.16
Industrial	NAA	Nitric and Adipic Acid Production	77.38%	80.71%	83.93%	85.70%	86.62%	87.48%	87.86%	88.23%	1.09
Industrial	EPS	Electric Power System	68.10%	68.69%	69.23%	69.73%	70.23%	70.70%	71.12%	71.51%	1.04
Industrial	FPD	Flat Panel Display Manufacturing	67.39%	67.59%	67.78%	67.96%	68.12%	68.28%	68.44%	68.58%	1.01
Energy	COL	Coal Mining Activities	63.47%	63.79%	63.81%	63.81%	63.88%	64.17%	64.39%	64.64%	1.01
Industrial	PV	Photovoltaic Cell Manufacturing	72.35%	72.52%	72.68%	72.83%	72.97%	73.11%	73.24%	73.36%	1.01
Industrial	MG	Magnesium Manufacturing	97.22%	97.36%	97.50%	97.62%	97.74%	97.85%	97.96%	98.06%	1.01
Waste	LAN	Landfilling of Solid Waste	48.62%	51.49%	52.20%	53.34%	53.24%	52.80%	51.92%	51.07%	0.99
Agriculture	RICE	Rice Cultivation	36.81%	36.46%	36.38%	35.86%	35.22%	34.44%	33.72%	32.92%	0.90
Agriculture	CROP	Cropland Management	3.66%	3.55%	3.49%	3.34%	3.33%	3.29%	3.25%	3.20%	0.90
Agriculture	LIVE	Livestock	9.58%	9.37%	9.17%	8.97%	8.77%	8.56%	8.36%	8.14%	0.87
Industrial	SEMI	Semiconductor Manufacturing	66.93%	46.54%	37.19%	29.39%	27.36%	25.51%	23.77%	22.06%	0.47

Supplementary Table 8 Zero-cost MAC reduction

**MAC control**	**No. of GCAM regions**	**Global averaged MAC reductions at no cost**
Foams	9	53%
Solvent Use	17	49%
Aerosols Product Use	17	48%
Natural Gas and Oil Systems	17	36%
Refrigeration and Air Conditioning	17	31%
Electric Power Systems	17	19%
Landfilling of Solid Waste	17	14%
Aluminum and Magnesium Production	16	13%
Coal Mining Activities	17	10%
Cropland Management	17	5%
Rice Cultivation	16	5%
Livestock	17	4%
Semiconductor Manufacturing	12	1%
Wastewater	17	0%
Fire Protection	17	0%
Flat Panel Display Manufacturing	3	0%
HCFC-22 Production	9	0%
Nitric and Adipic Acid Production	15	0%
Photovoltaic Cell Manufacturing	9	0%

Supplementary Table 9 Energy CH_4 emissions (Gt CO_2-eq yr^{-1}) for 32 GCAM regions in the Reference scenario. Emissions were aggregated with GWP-100 from ref[3].

**GCAM region**	**2015**	**2020**	**2050**	**2100**
Africa_Eastern	0.06	0.07	0.07	0.06
Africa_Northern	0.14	0.15	0.16	0.17
Africa_Southern	0.08	0.09	0.23	0.48
Africa_Western	0.20	0.21	0.44	1.01
Argentina	0.03	0.03	0.04	0.06
Australia_NZ	0.06	0.06	0.08	0.20
Brazil	0.08	0.09	0.09	0.08
Canada	0.07	0.06	0.09	0.15
Central America and Caribbean	0.03	0.04	0.05	0.06
Central Asia	0.20	0.20	0.19	0.17
China	0.77	0.72	0.45	0.32
Colombia	0.03	0.03	0.04	0.04
EU-12	0.07	0.09	0.06	0.05
EU-15	0.13	0.17	0.24	0.29
Europe_Eastern	0.07	0.08	0.11	0.14
Europe_Non_EU	0.04	0.04	0.05	0.04
European Free Trade Association	0.00	0.01	0.02	0.04
India	0.16	0.17	0.17	0.19
Indonesia	0.17	0.19	0.20	0.21
Japan	0.01	0.02	0.03	0.07
Mexico	0.08	0.09	0.10	0.13
Middle East	0.33	0.34	0.45	0.32
Pakistan	0.02	0.02	0.03	0.03
Russia	0.73	0.63	0.49	0.51
South Africa	0.03	0.03	0.06	0.06
South America_Northern	0.04	0.06	0.26	0.59
South America_Southern	0.05	0.05	0.06	0.06
South Asia	0.10	0.13	0.30	0.56
South Korea	0.01	0.02	0.01	0.01
Southeast Asia	0.18	0.20	0.30	0.37
Taiwan	0.01	0.01	0.01	0.01
USA	0.48	0.43	0.53	0.65

Supplementary Table 10 AgLanduse CH_4 emissions (Gt CO_2-eq yr^{-1}) for 32 GCAM regions in the Reference scenario. Emissions were aggregated with GWP-100 from ref[3].

**GCAM region**	**2015**	**2020**	**2050**	**2100**
Africa_Eastern	0.20	0.23	0.59	1.27
Africa_Northern	0.03	0.04	0.07	0.08
Africa_Southern	0.08	0.09	0.18	0.32
Africa_Western	0.23	0.26	0.53	0.99
Argentina	0.08	0.09	0.12	0.13
Australia_NZ	0.08	0.09	0.14	0.19
Brazil	0.31	0.32	0.38	0.39
Canada	0.03	0.03	0.04	0.06
Central America and Caribbean	0.04	0.04	0.05	0.06
Central Asia	0.07	0.07	0.11	0.12
China	0.57	0.62	0.70	0.50
Colombia	0.05	0.05	0.08	0.09
EU-12	0.04	0.04	0.05	0.06
EU-15	0.19	0.20	0.24	0.28
Europe_Eastern	0.02	0.02	0.03	0.04
Europe_Non_EU	0.04	0.04	0.05	0.06
European Free Trade Association	0.01	0.01	0.01	0.01
India	0.38	0.43	0.70	0.81
Indonesia	0.07	0.07	0.09	0.09
Japan	0.02	0.02	0.02	0.02
Mexico	0.05	0.05	0.07	0.08
Middle East	0.04	0.04	0.08	0.12
Pakistan	0.11	0.13	0.24	0.38
Russia	0.06	0.06	0.07	0.07
South Africa	0.02	0.03	0.04	0.04
South America_Northern	0.03	0.02	0.03	0.04
South America_Southern	0.09	0.10	0.12	0.14
South Asia	0.08	0.10	0.18	0.27
South Korea	0.01	0.02	0.02	0.01
Southeast Asia	0.23	0.24	0.31	0.33
Taiwan	0.00	0.00	0.00	0.00
USA	0.25	0.26	0.35	0.45

Supplementary Table 11 Energy N_2O emissions (Gt CO_2-eq yr^{-1}) for 32 GCAM regions in the Reference scenario. Emissions were aggregated with GWP-100 from ref[3].

**GCAM region**	**2015**	**2020**	**2050**	**2100**
Africa_Eastern	0.01	0.01	0.01	0.03
Africa_Northern	0.01	0.02	0.03	0.05
Africa_Southern	0.01	0.02	0.05	0.21
Africa_Western	0.02	0.02	0.03	0.05
Argentina	0.00	0.00	0.00	0.00
Australia_NZ	0.01	0.01	0.01	0.01
Brazil	0.01	0.01	0.02	0.02
Canada	0.01	0.01	0.01	0.01
Central America and Caribbean	0.00	0.00	0.01	0.01
Central Asia	0.01	0.01	0.01	0.01
China	0.19	0.20	0.23	0.15
Colombia	0.00	0.00	0.00	0.01
EU-12	0.01	0.01	0.01	0.01
EU-15	0.04	0.04	0.04	0.04
Europe_Eastern	0.01	0.01	0.01	0.01
Europe_Non_EU	0.01	0.01	0.02	0.02
European Free Trade Association	0.00	0.00	0.00	0.00
India	0.03	0.04	0.08	0.09
Indonesia	0.01	0.01	0.02	0.03
Japan	0.01	0.01	0.01	0.01
Mexico	0.01	0.01	0.01	0.01
Middle East	0.04	0.04	0.05	0.06
Pakistan	0.00	0.01	0.01	0.01
Russia	0.01	0.01	0.01	0.01
South Africa	0.00	0.00	0.01	0.01
South America_Northern	0.00	0.00	0.00	0.00
South America_Southern	0.00	0.00	0.01	0.01
South Asia	0.01	0.01	0.03	0.06
South Korea	0.01	0.01	0.01	0.00
Southeast Asia	0.02	0.03	0.04	0.05
Taiwan	0.00	0.00	0.00	0.00
USA	0.06	0.06	0.08	0.09

Supplementary Table 12 AgLanduse N_2O emissions (Gt CO_2-eq yr^{-1}) for 32 GCAM regions in the Reference scenario. Emissions were aggregated with GWP-100 from ref[3].

**GCAM region**	**2015**	**2020**	**2050**	**2100**
Africa_Eastern	0.11	0.13	0.26	0.46
Africa_Northern	0.05	0.06	0.09	0.10
Africa_Southern	0.07	0.08	0.15	0.24
Africa_Western	0.10	0.11	0.21	0.32
Argentina	0.08	0.09	0.11	0.13
Australia_NZ	0.02	0.03	0.05	0.05
Brazil	0.15	0.16	0.22	0.24
Canada	0.03	0.03	0.05	0.06
Central America and Caribbean	0.04	0.04	0.06	0.07
Central Asia	0.03	0.03	0.04	0.05
China	0.44	0.47	0.53	0.42
Colombia	0.04	0.04	0.06	0.06
EU-12	0.04	0.04	0.05	0.05
EU-15	0.14	0.15	0.18	0.20
Europe_Eastern	0.04	0.04	0.05	0.06
Europe_Non_EU	0.03	0.04	0.04	0.05
European Free Trade Association	0.00	0.00	0.01	0.01
India	0.08	0.09	0.13	0.15
Indonesia	0.04	0.04	0.06	0.06
Japan	0.01	0.01	0.01	0.01
Mexico	0.03	0.03	0.04	0.05
Middle East	0.05	0.06	0.11	0.12
Pakistan	0.02	0.02	0.03	0.03
Russia	0.08	0.08	0.11	0.12
South Africa	0.02	0.02	0.02	0.03
South America_Northern	0.02	0.02	0.03	0.03
South America_Southern	0.14	0.14	0.14	0.16
South Asia	0.04	0.05	0.08	0.11
South Korea	0.01	0.01	0.01	0.01
Southeast Asia	0.08	0.08	0.12	0.13
Taiwan	0.00	0.00	0.00	0.00
USA	0.31	0.34	0.46	0.54

Supplementary Table 13 HFCs emissions (Gt CO_2-eq yr^{-1}) for 32 GCAM regions in Reference scenario. Emissions were aggregated with GWP-100 from ref[3].

**GCAM region**	**2015**	**2020**	**2050**	**2100**
Africa_Eastern	0.00	0.00	0.03	0.22
Africa_Northern	0.02	0.02	0.07	0.09
Africa_Southern	0.00	0.00	0.02	0.15
Africa_Western	0.02	0.02	0.13	1.24
Argentina	0.02	0.02	0.03	0.04
Australia_NZ	0.01	0.01	0.02	0.02
Brazil	0.02	0.02	0.07	0.09
Canada	0.01	0.01	0.01	0.01
Central America and Caribbean	0.01	0.01	0.04	0.07
Central Asia	0.00	0.00	0.01	0.02
China	0.21	0.30	0.59	0.40
Colombia	0.00	0.00	0.01	0.02
EU-12	0.01	0.01	0.02	0.01
EU-15	0.08	0.09	0.10	0.09
Europe_Eastern	0.00	0.00	0.00	0.00
Europe_Non_EU	0.01	0.01	0.02	0.02
European Free Trade Association	0.00	0.00	0.00	0.00
India	0.01	0.02	0.24	0.77
Indonesia	0.01	0.03	0.17	0.22
Japan	0.02	0.02	0.02	0.02
Mexico	0.02	0.02	0.04	0.09
Middle East	0.12	0.15	0.32	0.39
Pakistan	0.01	0.01	0.06	0.14
Russia	0.01	0.01	0.02	0.03
South Africa	0.01	0.01	0.02	0.03
South America_Northern	0.01	0.00	0.01	0.02
South America_Southern	0.00	0.00	0.01	0.02
South Asia	0.00	0.01	0.05	0.13
South Korea	0.03	0.03	0.04	0.03
Southeast Asia	0.05	0.07	0.22	0.34
Taiwan	0.00	0.01	0.02	0.02
USA	0.14	0.15	0.18	0.18

Supplementary Table 14 PFCs emissions (Gt CO_2-eq yr^{-1}) for 32 GCAM regions in Reference scenario. Emissions were aggregated with GWP-100 from ref[3].

**GCAM region**	**2015**	**2020**	**2050**	**2100**
Africa_Eastern	0.00	0.00	0.00	0.00
Africa_Northern	0.00	0.00	0.00	0.00
Africa_Southern	0.00	0.00	0.00	0.00
Africa_Western	0.00	0.00	0.00	0.00
Argentina	0.00	0.00	0.00	0.00
Australia_NZ	0.00	0.00	0.00	0.00
Brazil	0.00	0.00	0.00	0.00
Canada	0.00	0.00	0.00	0.00
Central America and Caribbean	0.00	0.00	0.00	0.00
Central Asia	0.02	0.03	0.03	0.02
China	0.00	0.00	0.00	0.00
Colombia	0.00	0.00	0.00	0.00
EU-12	0.00	0.00	0.00	0.00
EU-15	0.00	0.00	0.00	0.00
Europe_Eastern	0.00	0.00	0.00	0.00
Europe_Non_EU	0.00	0.00	0.00	0.00
European Free Trade Association	0.00	0.00	0.00	0.00
India	0.00	0.00	0.00	0.00
Indonesia	0.00	0.00	0.00	0.00
Japan	0.00	0.00	0.00	0.00
Mexico	0.00	0.00	0.01	0.01
Middle East	0.00	0.00	0.00	0.00
Pakistan	0.00	0.00	0.00	0.00
Russia	0.00	0.00	0.00	0.00
South Africa	0.00	0.00	0.00	0.00
South America_Northern	0.00	0.00	0.01	0.01
South America_Southern	0.01	0.01	0.01	0.01
South Asia	0.00	0.00	0.00	0.00
South Korea	0.00	0.00	0.00	0.00
Southeast Asia	0.00	0.00	0.00	0.00
Taiwan	0.00	0.00	0.00	0.00
USA	0.00	0.00	0.00	0.00

Supplementary Table 15 SF_6 emissions (Gt CO_2-eq yr^{-1}) for 32 GCAM regions in Reference scenario. Emissions were aggregated with GWP-100 from ref[3].

**GCAM region**	**2015**	**2020**	**2050**	**2100**
Africa_Eastern	0.00	0.00	0.00	0.02
Africa_Northern	0.00	0.00	0.01	0.01
Africa_Southern	0.00	0.00	0.00	0.02
Africa_Western	0.00	0.00	0.01	0.04
Argentina	0.00	0.00	0.00	0.00
Australia_NZ	0.00	0.00	0.00	0.00
Brazil	0.00	0.00	0.00	0.00
Canada	0.00	0.00	0.00	0.00
Central America and Caribbean	0.00	0.00	0.00	0.00
Central Asia	0.00	0.00	0.00	0.00
China	0.03	0.04	0.05	0.04
Colombia	0.00	0.00	0.00	0.00
EU-12	0.00	0.00	0.00	0.00
EU-15	0.00	0.00	0.00	0.00
Europe_Eastern	0.00	0.00	0.00	0.00
Europe_Non_EU	0.00	0.00	0.00	0.01
European Free Trade Association	0.00	0.00	0.00	0.00
India	0.01	0.01	0.03	0.05
Indonesia	0.00	0.00	0.00	0.01
Japan	0.00	0.00	0.00	0.00
Mexico	0.00	0.00	0.00	0.01
Middle East	0.01	0.01	0.02	0.03
Pakistan	0.00	0.00	0.00	0.01
Russia	0.00	0.00	0.00	0.00
South Africa	0.00	0.00	0.00	0.00
South America_Northern	0.00	0.00	0.00	0.00
South America_Southern	0.00	0.00	0.00	0.00
South Asia	0.00	0.00	0.00	0.01
South Korea	0.01	0.01	0.01	0.01
Southeast Asia	0.00	0.00	0.01	0.02
Taiwan	0.01	0.01	0.01	0.01
USA	0.00	0.00	0.00	0.02

Supplementary Table 16 End-of-century global mean temperature change for all 1.5°C pathways with different net-zero CO_2 years, paired with different levels of non-CO_2 GHG mitigation levels. In 1.5°C pathways, CO_2 emissions linearly reduce to -8 $GtCO_2$ yr^{-1} and stay constant. Values in bold indicate scenarios reaching 1.5°C targets.

Net-zero year	*CO_2 abatement only*	*CO_2-driven GHG abatement*	*Comprehensive GHG abatement*
2028	1.68	**1.44**	**1.14**
2032	1.73	**1.50**	**1.20**
2037	1.79	1.57	**1.27**
2041	1.85	1.62	**1.33**
2045	1.91	1.67	**1.37**
2049	1.97	1.73	**1.43**
2053	2.02	1.79	**1.50**
2057	2.08	1.86	1.56
2061	2.14	1.92	1.62
2065	2.20	1.99	1.69
2070	2.26	2.05	1.76
2074	2.32	2.12	1.83
2078	2.38	2.19	1.90
2082	2.44	2.26	1.97
2086	2.50	2.33	2.04

Supplementary Table 17 End-of-century global mean temperature change for all 2°C pathways with different net-zero CO_2 years, paired with different non-CO_2 GHG mitigation levels. In 2°C pathways, CO_2 emissions linearly reduce to 0 $GtCO_2$ yr^{-1} and stay constant. Values in bold indicate scenarios reaching well-below 2°C targets.

Net-zero year	*CO_2 abatement only*	*CO_2-driven GHG abatement*	*Comprehensive GHG abatement*
2030	**1.99**	**1.82**	**1.51**
2035	2.04	**1.86**	**1.56**
2040	2.08	**1.91**	**1.60**
2045	2.13	**1.95**	**1.65**
2050	2.17	2.00	**1.70**
2055	2.22	2.05	**1.75**
2060	2.27	2.10	**1.80**
2065	2.31	2.15	**1.85**
2070	2.36	2.20	**1.90**
2075	2.41	2.25	**1.95**
2080	2.46	2.30	2.00
2085	2.50	2.35	2.06
2090	2.55	2.41	2.12
2095	2.60	2.47	2.17
2100	2.64	2.52	2.23

Supplementary Table 18 Non-CO_2 emission and climate forcing changes in mitigation scenarios relative to Reference in 2050 and 2100 when CO_2 emissions reach net-zero by 2075 under 2°C pathway (percentage reductions are shown in parentheses). Non-CO_2 GHG emissions were aggregated with GWP-100 from ref[3].

Scenario	CH_4 Energy	CH_4 AgLanduse	N_2O Energy	N_2O AgLanduse	HFCs	PFCs	SF_6
**2050 Emission (Gt CO_2-eq yr^{-1})**							
CO_2-driven	-1.20 (-22.2%)	-0.33 (-5.9%)	-0.21 (-24.9%)	-0.15 (-4.4%)	-0.14 (-5.4%)	-0.01 (-11.0%)	0.02 (10.4%)
Comprehensive	-2.61 (-48.2%)	-0.88 (-15.5%)	-0.47 (-55.1%)	-0.52 (-14.7%)	-1.71 (-66.0%)	-0.03 (-43.4%)	-0.09 (-50.5%)
**2100 Emission (Gt CO_2-eq yr^{-1})**							
CO_2-driven	-3.36 (-47.2%)	-1.25 (-16.7%)	-0.54 (-49.4%)	-0.68 (-16.6%)	-0.19 (-3.8%)	-0.01 (-14.6%)	0.10 (33.3%)
Comprehensive	-5.64 (-79.4%)	-1.91 (-25.5%)	-0.86 (-79.2%)	-1.16 (-28.1%)	-4.51 (-91.6%)	-0.06 (-78.9%)	-0.20 (-67.9%)
**2050 Forcing (W m^{-2})**							
CO_2-driven	-0.034 (-8.4%)	0.000 (0.1%)	-0.006 (-4.3%)	0.000 (0.2%)	-0.006 (-4.8%)	0.000 (-1.3%)	0.000 (1.2%)
Comprehensive	-0.105 (-25.4%)	-0.017 (-8.1%)	-0.017 (-12.2%)	-0.004 (-3.3%)	-0.055 (-40.9%)	0.000 (-5.2%)	-0.001 (-21.1%)
**2100 Forcing (W m^{-2})**							
CO_2-driven	-0.128 (-24.2%)	-0.021 (-7.7%)	-0.036 (-15.9%)	-0.015 (-8.3%)	-0.017 (-4.9%)	-0.001 (-4.6%)	0.003 (19.2%)
Comprehensive	-0.255 (-48.4%)	-0.055 (-20.4%)	-0.068 (-30.2%)	-0.032 (-18.0%)	-0.286 (-84.6%)	-0.002 (-19.6%)	-0.008 (-46.1%)

References

1 EPA, U. Global Non-CO2 Greenhouse Gas Emission Projections & Mitigation 2015-2050. (United States Environmental Protection Agency, Washington, DC 20005, 2019).

2 US EPA. Global Mitigation of Non-CO2 Greenhouse Gases: 2010-2030. *EPA-430-R-13-011, Washington, DC 20005* (2013).

3 Intergovernmental Panel On Climate Change. Climate change 2007: The physical science basis. *Agenda* **6**, 333 (2007).

4 Pebesma, E. Simple Features for R: Standardized Support for Spatial Vector Data. *The R Journal* **10**, 439-446, doi:10.32614/RJ-2018-009 (2018).

5 Ou, Y. Source data used to reproduce global maps for Ou et al. 2021 (Deep Mitigation of CO2 and non-CO2 Greenhouse Gases towards 1.5°C and 2°C Futures) [Data set]. *Zenodo.* https://doi.org/10.5281/zenodo.5484428 (2021).
