# Supplementary figures and images for: Deep mitigation of CO2 and non-CO2 greenhouse gases toward 1.5 °C and 2 °C futures
Source: Nat Commun. 2021 Oct 29;12:6245. doi: 10.1038/s41467-021-26509-z (PMC8556229; doi:10.1038/s41467-021-26509-z)

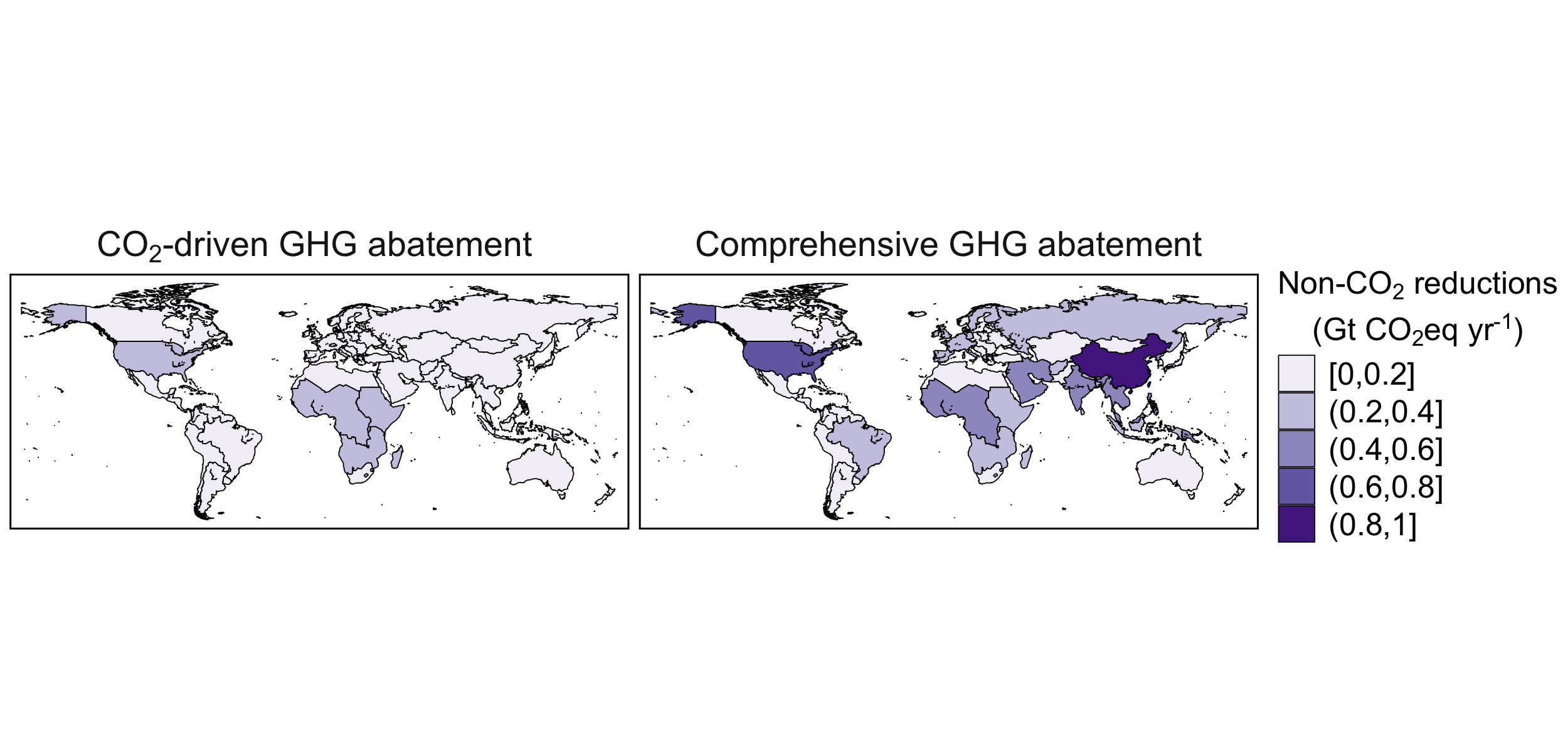

Supplement: Supplementary file 2 — Source Data [file 41467_2021_26509_MOESM2_ESM.zip › Map_reproduce/Fig 3d nonCO2 reductions 2050.png]
